# Whole genome and transcriptome analyses identify genetic markers associated with growth traits in Qinchuan black pig

**DOI:** 10.1186/s12864-025-11627-5

**Published:** 2025-05-12

**Authors:** Yaxin Wang, Guangquan Lv, Zhe Liu, Ye Cheng, Rongrong Ding, Gongshe Yang, Taiyong Yu

**Affiliations:** Key Laboratory of Animal Genetics, Breeding and Reproduction of Shaanxi Province, Laboratory of Animal Fat Deposition and Muscle Development, College of Animal Science and Technology, Northwest A&F University, Yangling, Shaanxi 712100 China

**Keywords:** Qinchuan black pig, Selection signal analysis, Genome-wide association studies, Differential expression analysis, Growth traits, Candidate genes

## Abstract

**Background:**

Growth traits are economically important traits in pig breeding. However, the genetic mechanism of growth traits is still unclear. Qinchuan Black (QCB) pigs are crossbred and produced by hybridizing Guanzhong Black (GZB) pigs and Large White (LW) pigs, its characteristics include fast growth and excellent meat quality. In this study, whole genome and transcriptome analyses revealed the candidate genes associated with growth traits in QCB pigs based on imputed low-coverage whole-genome resequencing data.

**Results:**

In total, we used 197 low-depth whole-genome resequencing data with an average depth of 3.5X, and then the data were imputed to resequencing data using SWIM reference panel, the imputation accuracy parameters, allele frequency r^2^ and concordance rate were 0.86 and 95.83%, respectively. We used two methods to investigate the candidate genes affecting the growth traits of QCB pigs, a total of 371 PSGs were identified, which related to muscle tissue development, tissue development and system development. A total of 30,489,782 SNPs were retained. A GWAS of ten growth traits by using fixed and random model circulating probability unification (FarmCPU) model, was performed in QCB pigs. We discovered seven genome wide significant SNPs and eight genome wide suggestive significant SNPs associated with body weight at 2 months (2-BW), body length at 2 months (2-BL), body height at 2 months (2-BH) and body height at 4 months (4-BH), and eighteen potential candidate genes were discovered. Transcriptomic data revealed that 18 differentially expression genes related to muscle and growth and development. Additionally, whole genome and transcriptome analyses found six genes (*TENM3*, *CTNND2*, *RIMS1*, *PCDH7*, *ADGRL3* and *CTNNA3*) may affect the growth traits in Qinchuan Black pigs.

**Conclusion:**

Our study shows that more candidate genes associated with pig growth traits can be identified by whole genome and transcriptome analyses. We found that six genes may be new key candidate genes affecting pig growth traits. In conclusion, this study elucidated the molecular genetic mechanisms of growth traits and identified new molecular breeding targets, offering a robust scientific basis for advancing breeding strategies and genetic investigations within this breed.

**Supplementary Information:**

The online version contains supplementary material available at 10.1186/s12864-025-11627-5.

## Introduction

Pigs these days are significant agricultural animals with a long history of domestication and economic importance [1]. From early domestication to modern breeding practices, artificial selection for agricultural economic traits has shaped the genomes of domestic pigs and led to many breeds and populations worldwide [2]. Growth traits such as live backfat thickness (LBT), average daily gain (ADG), body length (BL), body height (BH), chest circumference (CC), and tube circumference (TC) play a crucial role in porcine breeding programs and overall pig production [3, 4]. The body character index is commonly used as a direct indicator of production in pig breeding [5]. Various factors, including genetic and non-genetic influences such as pig breed, feeding behavior, and nutrition levels, impact the growth traits of pigs [6, 7].

While traditional breeding methods have improved pig growth performance over the years, growth traits are complex quantitative traits controlled by a combination of major and minor genes, making conventional breeding methods limited in their effectiveness. With advancements in molecular markers and the completion of the pig genome sequence, molecular breeding has emerged as an efficient approach to enhancing growth traits. As of August 25, 2024, the pig QTL database (https://www.animalgenome.org/cgi-bin/QTLdb/SS/summary) has documented 4,087 QTL related to growth traits. These discoveries have contributed a significant number of molecular markers to porcine breeding for growth traits.

The advancement of sequencing technology, coupled with its declining costs, has enabled numerous researchers to utilize low-depth resequencing for conducting relevant studies [8]. However, the sequencing quality is too low. Genotype imputation is a highly effective approach in genome-wide association studies (GWAS) [9], widely employed in human genetics research [10, 11]. This method enhances the quantity and density of SNPs available for association analysis, thereby enabling the identification of novel candidate genes. In addition, selection signatures in the genome have been used frequently to understand the relationships between genotype and phenotype in pigs. For instance, strong selection signatures were found at three loci which were related to morphological changes in the domestic pigs using whole-genome resequencing [12]; evidence of artificial selection of lean muscle mass, fertility and immunization traits were revealed in Duroc pigs [13].

Recently, with the development of high-throughput sequencing technology, the genome and transcriptome technologies has become an important means and routine to analyze the molecular mechanism of agricultural complex traits in farm animals [14]. In this study, whole genome and transcriptome analyses identified candidate genes associated with growth traits in QCB pigs based on imputed low-depth whole-genome resequencing data. This result will understand the QCB pig’s genome characteristics, and provide a molecular basis for accelerating the breeding process of QCB pigs.

## Materials and methods

### Ethics statement

The study was approved by the Institutional Animal Care and Use Committee of Northwest A&F University (Yangling, China), and all operations were carried out according to the university’s guidelines for animal research. All pigs were cared for and slaughtered according to the guidelines of the Institutional Animal Care and Use Committee of Northwest A&F University (Yangling, China) [15].

### Animals and phenotype

The pig population at the core breeding farm of Northwest A&F University (Yangling, China) was reared uniformly. Breeding information and lineage records of Qinchuan black (QCB) pigs from 2022 to 2023 were collected. A total of 197 QCB pigs with complete pedigrees were included in the study. We collected ten growth traits, mainly include two months and four months body weight, body length, body height, chest circumference, and tube circumference. Body length was measured from the midpoint of the ear to the tail, body height from shoulder to ground, chest circumference by circling the trailing edge of the scapula, and tube circumference at the upper third of the anterior tube of the pig. All measurements were taken on a flat surface with the pig in a natural standing posture.

### Estimation of genetic parameters and genetic correlations

The variance and covariance components, along with genetic correlations for the ten traits, were estimated using ASREML (version 4.10) [16] software. The following animal model was applied:

$$\:\varvec{Y}=\varvec{X}\varvec{b}+\varvec{Z}1\varvec{a}+\varvec{Z}2\varvec{c}+\varvec{e}$$

In the model, Y represents the vector of phenotypic records; b denotes the vector of fixed effects, including farrowing year, farrowing season, gender, and parity; X is the design matrix linking b to Y; a is the vector of additive genetic effects; c is the vector of maternal effects; e is the vector of random residual effects; X, Z1, and Z2 are the corresponding incidence matrices for fixed effects, additive genetic effects, and maternal effects, respectively.

The genetic correlation was calculated as follows:

$$r12 = \frac{{cov\left( {a1{\text{}},{\text{}}a2} \right)}}{{\sigma a1\sigma a2}}$$

r12 is the genetic correlation between trait 1 and trait 2; a1 and a2 represent the additive genetic values of trait 1 and trait 2 for the same individuals; cov (a1, a2) is the genetic covariance between the two traits; σa1, and σa2 are the genetic standard deviations of trait 1 and trait 2, respectively.

### Data set collection and generation

In this study, we collected ten growth traits data and ear tissue samples from third-generation 197 QCB pigs at the QCB Pig Breeding Farm of the Animal Husbandry Teaching and Experiment Base of Northwest A&F University, and extracted genomic DNA from these samples. The concentration and quality of the extracted DNA samples were measured using a NanoDrop2000 spectrophotometer, with the DNA quality range requiring an A260/280 ratio between 1.7 and 2.2, an A260/230 ratio between 1.8 and 2.2, and ensuring a DNA concentration greater than 50 ng/µL. This study was based on low-depth whole-genome resequencing conducted by BGI Genomics (Shenzhen) using their DNB sequencing platform, with a sequencing depth of approximately 3.5X for each individual. Furthermore, a total of 212 WGS-seq samples were collected from our previous sequenced and NCBI Sequence Read Archive, which including 11 wild boar breeds and 31 domestic pig breeds (Table S1).

### Genotype imputation pipeline

Swine Imputation (SWIM 1.0) [17] reference panel is the reference panel for pigs, including 30,489,782 single nucleotide polymorphisms (SNPs) and 4,125,579 insertions/deletions (indels). Due to the average sequencing depth being only 3.5X, we employed the SWIM reference panel with default parameter settings to perform genotype imputation, bridging the target and reference genotype data. We extracted 3.5X from 97 F2 generation QCB pigs with 10X high-depth resequencing to evaluate the accuracy before and after imputation. Additionally, because the common low-depth sequencing is 0.5X-1X, we also extracted 1X from the 97 F2 generation QCB pigs with 10X high-depth resequencing to evaluate the accuracy before and after imputation. We performed genotype imputation on the extracted 1X and 3.5X bam files using the SWIM reference panel via the QUILT (v1.0.5) [18] software, which the parameters are “--output dir --chr --region start --region end --buffer --bam list --sample names_file --output_filename”. We evaluated imputation accuracy using two widely accepted metrics: concordance rate, non-reference concordance rate, and r². The concordance rate is calculated as the percentage of individuals whose imputed genotypes match their observed genotypes. The r² metric represents the squared Pearson correlation coefficient between the observed and imputed genotypes. We calculated these metrics on a per-SNP basis and then averaged them across SNPs within minor allele frequency (MAF) bins or across the entire genome. After imputing, the 1X and 3.5X mean allelic concordance rate and squared correlation (r^2^) was 95.55%, 95.83%, 0.85 and 0.86, respectively. The results showed a significant improvement in the accuracy of the 3.5X after imputation compared to the 1X.

### Genotype imputation of low depth sequencing data

The quality control for the 197 low-depth resequencing data was performed using fastp (v.0.20.1) [19] with default settings. The high-quality reads were aligned to the Sscrofa11.1 reference sequence using the Burrows-Wheeler Aligner (BWA) software (v.0.7.8) with the parameters of “mem-t 10-k 32-M”. We then converted the mapping reads into bam file and sorted the files using SAMtools (v.1.10) [20]. Then we used the SWIM reference panel to impute the 197 low-depth resequencing individuals via the QUILT (v1.0.5) software. After imputation, the VCF files of the 18 autosomes generated were merged for subsequent analysis.

### Variation calling and annotation

The quality control for the 212 WGS-seq data was performed using fastp (v.0.20.1) [19] with default settings. The high-quality reads were aligned to the Sscrofa11.1 reference sequence using the Burrows-Wheeler Aligner (BWA) software (v.0.7.8) with the parameters of “mem-t 10-k 32-M”. We then converted the mapping reads into bam file and sorted the files using SAMtools (v.1.10) [20]. Duplicates were removed by the MarkDuplicates module in GATK (v.4.2.6.1) [21]. SNPs were called from the bam files by the GATK HaplotypeCaller module with the GATK best-practice recommendations. Raw GVCFs with the samples called individually were merged using the GenomicsDBImport and converted for SNPs into variants files using GenotypeGVCFs. We then selected the candidate SNPs created the selected SNPs data using the GATK module SelectVariants, which generated genotype calls in Variant Call Formats (VCF). To exclude possible false positives, we filtered the variants according to the strict filter criteria. High-quality SNPs were identified according to the filtering criteria: QUAL > 30.0, OD > 5.0, FS < 60.0, MQ > 40.0, MQRankSum > − 12.5, ReadPosRankSum > − 8.075.

### Population genetic structure analysis and linkage disequilibrium

We used Bcftools (v.1.17) [19] to merge the two VCF files to produce a single VCF file that had 410 samples. The VCF file was quality controlled using PLINK (v.1.90) [22], and Minimal Allele Frequencies (MAF) less than 0.01, genotype detection rate less than 0.1, and Hardy-Weinberg equilibrium (HWE) less than 0.001 were excluded. PLINK (v.1.90) was used to calculate the average share allele distance matrix between individuals (--distance-matrix). The result of tree construction was displayed using MEGA11 [23] and iTOL [24]. Population structure was conducted by the program ADMIXTURE (v.1.3.0) [25] with the default settings. To reveal the relationships among the Eurasian pigs, a principal component analysis (PCA) was performed using PLINK (v.1.90) and plotted by in-house R scripts. We examined the patterns of LD decay within each species or population by random selection of three individuals to avoid biases by difference in sample sizes and using the Vcftools v0.1.16 to extract individual data for LD analysis. Pairwise LD estimates were measured as parameter r^2^ with a maximum distance of 500 kb using the PopLDdecay v.3.4.1 [26].

### Analysis of genome‑wide selective sweep regions

Selection signatures analysis was performed by constructing genetic variation databases of QCB pigs and LW pigs with quality control using the parameters --geno 0.1 --maf 0.05 --hwe 0.001. To identify candidate regions under positive selection in QCB pigs, we initially computed fixation statistics (Fst) and population nucleotide diversity ratio (θπ) after a specified procedure. Using VCFtools (v.0.1.16), we calculated average Fst and θπ in 50 kb sliding windows with a 25 kb step size, comparing QCB pigs with two control breeds. The top 5% ranked windows based on Fst and θπ scores were identified as candidate selective regions. Subsequently, after annotation with Annovar software, common genes selected through both analyses were considered as positively selected genes.

### Single-locus GWAS

The imputation VCF file of 197 individuals were quality controlled for all individual genotype data using PLINK v1.90 with the quality control parameters: --geno 0.05 --maf 0.05 --hwe 1e-6, and the --indep --pairwise 100 10 0.5 parameter, LD trimming was performed, and finally 18 autosomes totaling 540,759 SNPs were retained for GWAS. We used FarmCPU model for GWAS in GAPIT3 [27]. FarmCPU is a statistical model used for GWAS. In the FarmCPU model, fixed effects are typically used to describe the impact of known, controllable genetic variations on phenotypes, while random effects are employed to capture the influence of unknown, uncontrollable genetic variations on phenotypes. By integrating fixed and random effects, the FarmCPU model can more accurately identify genetic variations associated with phenotypes and reduce the occurrence of false positive results. In this study, candidate SNPs were identified using the Bonferroni correction method, with *P* < 1/N and *P* < 0.05/N representing the genome-wide suggestive and significance thresholds, respectively. Visualization of the results, including Manhattan plots and Q-Q plots, was performed using the R CMplot package (version 4.2.0) [28].

### Multi-locus GWAS

FASTmrEMMA multi-locus GWAS approaches were employed using the R package “mrMLM”. The multi-locus approach is divided into two stages. In the first step, SNPs effects were treated as random; a small number of SNPs were selected based on the prior premise that most SNPs should have no effect on the quantitative traits. In the second step, all selected SNPs in the first step were placed into multi-locus model. Among the multi-locus GWAS approach, all parameters were set at default values except for the critical P value in the first step. In the first step, the critical P value was set at 0.005 for FASTmrEMMA [29]. It is worth mentioning that the critical LOD scores of all models are set to 3.0 in the second step.

### Candidate gene search

Candidate genes were identified using BedTools [30] by scanning regions 0.5 Mb upstream and downstream of the significant SNPs, aligned to the pig reference genome.

### Differential genes expressed analysis

In this study, transcriptome data from six QCB pig and six small pig longissimus dorsi muscles, the small pig longissimus dorsi muscles were downloaded from the NCBI SRA: PRJNA309102 and PRJNA716984 and analyzed for differentially expressed genes. To process transcriptome sequence data, fastp (v0.23.2) was used for quality filtering of the sequencing reads [19]. HISAT2 (v2.2.1) was used for a fast and accurate sequence aligned to the Sscrofa11.1 reference genome [31]. Finally, a transcriptome gene expression count file was converted using Samtools (v1.15.1) and featureCounts (v2.0.3) to obtain the gene expression profile in each sample [20]. And differentially expressed genes were identified by DESeq2 (v.1.20) [32]. Genes with a corrected p-value < 0.05 and fold changes > 2 or < 0.5 were assigned as significantly differential expressed.

### Functional enrichment analyses

To enhance understanding of the biological processes and pathways related to the candidate genes, GO terms enrichment analyses and KEGG pathway analysis were conducted using g: Profiler. Terms with a *P*-value less than 0.05 were deemed statistically significant [33].

## Results

### Descriptive statistics and genetic parameters estimations

**Fig. 1:**
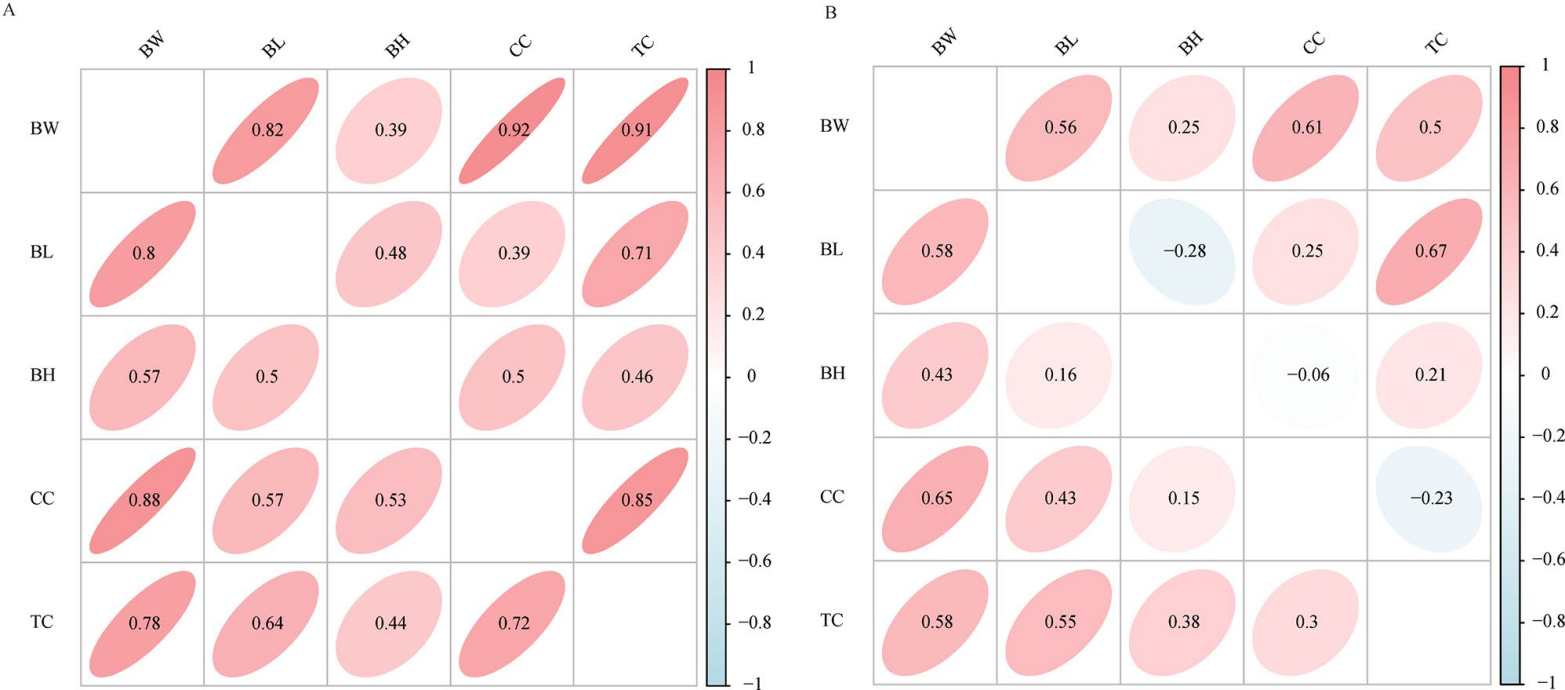
Phenotypic and genetic correlations between growth traits in Qinchuan black (QCB) pigs. (**A**) Phenotypic and genetic correlations between growth traits in QCB pigs at two months. (**B**) Phenotypic and genetic correlations between growth traits in QCB pigs at four months. The upper triangle represents genetic correlation, the lower triangle represents phenotypic correlation, and the numbers in the ellipse represent specific correlation coefficients. Body weight (BW), Body length (BL), Body height (BH), Chest circumference (CC), and Tube circumference (TC)

The maximum, minimum, mean, standard deviation, coefficient of variation (CV), and heritability of the ten growth traits are shown in Table 1. The mean of body weight at 2 months (2-BW), body length at 2 months (2-BL), body height at 2 months (2-BH), chest circumference at 2 months (2-CC), and tube circumference at 2 months (2-TC) and body weight at 4 months (4-BW), body length at 4 months (4-BL), body height at 4 months (4-BH), chest circumference at 4 months (4-CC), and tube circumference at 4 months (4-TC) were 10.67, 50.18, 32.58,48.95, 9.67, 26.74, 64.22, 44.07, 65.63 and 11.91, respectively. Except for 2-BW and 4-BW with a CV of 20%, the CV of other growth parameters (2-BL, 2-BH, 2-CC, 2-TC, 4-BL, 4-BH, 4-CC, 4-TC) were close to 10%. The heritability of growth parameters ranged from 0.0293 to 0.5049, among which 4-CC had the lowest heritability of 0.0293 and 2-CC had the greatest heritability of 0.5049. As can be seen, these traits are all medium-heritability traits. The phenotypic and genetic correlations of the growth parameters are presented in Fig. 1. For two months, the results showed a significant positive correlation between BW and BL, BW and BH, BW and CC, BW and TC, BL and BH, BL and CC, BL and TC, BH and CC, BH and TC and CC and TC (Fig. 1A). For four months, the results showed a significant positive correlation between BW and BL, BW and BH, BW and CC, BW and TC, BL and CC, BL and TC, BH and CC, BH and TC and CC and TC, whereas a significant negative correlation was observed between BL and BH, BH and CC, and CC and TC (Fig. 1B).

**Table 1:** Descriptive statistics of growth traits

Traits	Numbers	Max	Min	Mean	SD	CV^1^ (%)	Heritability
2-BW^2^(kg)	460	16.65	4.05	10.67	2.19	20.52%	0.43
2-BL^3^(cm)	460	63.00	35.50	50.18	4.55	9.07%	0.29
2-BH^4^(cm)	460	39.50	25.00	32.58	2.65	8.13%	0.24
2-CC^5^(cm)	460	60.50	37.00	48.95	4.07	8.31%	0.50
2-TC^6^(cm)	460	11.50	7.00	9.67	0.68	7.03%	0.25
4-BW^7^(kg)	282	43.35	13.40	26.74	5.40	20.19%	0.43
4-BL^8^(cm)	282	83.50	48.00	64.22	5.64	8.78%	0.25
4-BH^9^(cm)	282	51.00	35.30	44.07	3.25	7.37%	0.03
4-CC^10^(cm)	282	78.50	52.10	65.63	4.79	7.30%	0.03
4-TC^11^(cm)	282	14.00	10.00	11.91	0.77	6.47%	0.39

^1^CV: coefficient of variation; ^2^2-BW: body weight at 2 months, ^3^2-BL = body length at 2 months, ^4^2-BH = body height at 2 months; ^5^2-CC = chest circumference at 2 months; ^6^2-TC = tube circumference at 2 months; ^7^4-BW = body weight at 4 months; ^8^4-BL = body length at 4 months; ^9^4-BH = body height at 4 months; ^10^4-CC = chest circumference at 4 months; ^11^4-TC = tube circumference at 4 months

### Accuracy of genotype imputation

In this study, we extracted 1X and 3.5X from 97 F2 generation QCB pigs with 10X high-depth resequencing to evaluate the accuracy before and after imputation. Through evaluation, we found that the 1X and 3.5X mean allelic concordance rate and squared correlation (r^2^) was 60.85%, 83.96%, 0.13 and 0.52, respectively. After imputed by SWIM, the 1X and 3.5X mean allelic concordance rate and squared correlation (r^2^) was 95.55%, 95.83%, 0.85 and 0.86, respectively (Fig. S1).

### Population genetic structure and linkage disequilibrium

**Fig. 2:**
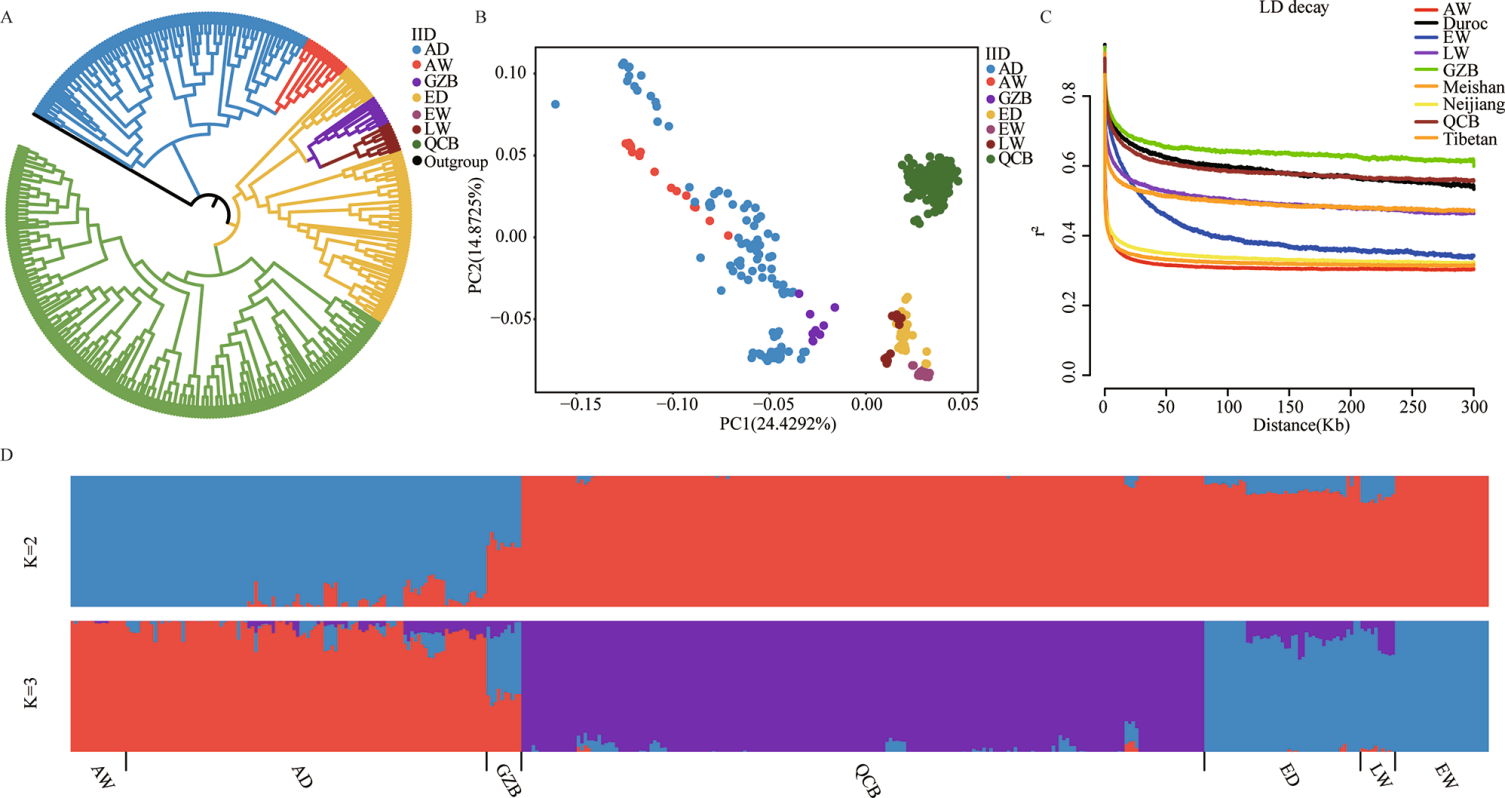
Phylogenetic relationship and population structure of QCB pigs and other six breeds tested in this study. (**A**) Neighbor-joining phylogenetic tree constructed from SNP data among seven populations. (**B**) PCA plot of population structure showing the top two principal components. (**C**) ADMIXTURE analysis with seven populations (K = 2-3). (**D**) Represents the linkage disequilibrium decay plot for nine pig breeds. Asian domestic (AD), Asian wild (AW), Guanzhong black (GZB), European domestic (ED), European wild (EW), Large white (LW), and Qinchuan black (QCB)

To assess the population structure and linkage disequilibrium of QCB pigs, we used the hard quality control criteria described in methods. After quality control, 5,034,676 SNPs were used to conduct population structure and linkage disequilibrium analyses. We examined the NJ tree of Asian pigs and European pigs and found that the two populations formed their own separate clusters. Meanwhile, we found that QCB pigs were located in European pig clusters (Fig. 2A). Then, we performed PCA analysis and found that QCB pigs, Asian pigs and European pigs were effectively separated. PC1 and PC2 explained approximately 24.4292% and 14.8725% of the total genetic variation, respectively (Fig. 2B). The breeds also indicated clearly separated clusters according to their geographical locations (Fig. 2D). Moreover, through analysis of LD, we discovered that a lower LD decay in QCB pigs than LW pigs, indicating that the selection caused the enhancement of LD degree in QCB pigs (Fig. 2C).

### Identification of selection signatures in QCB

**Fig. 3:**
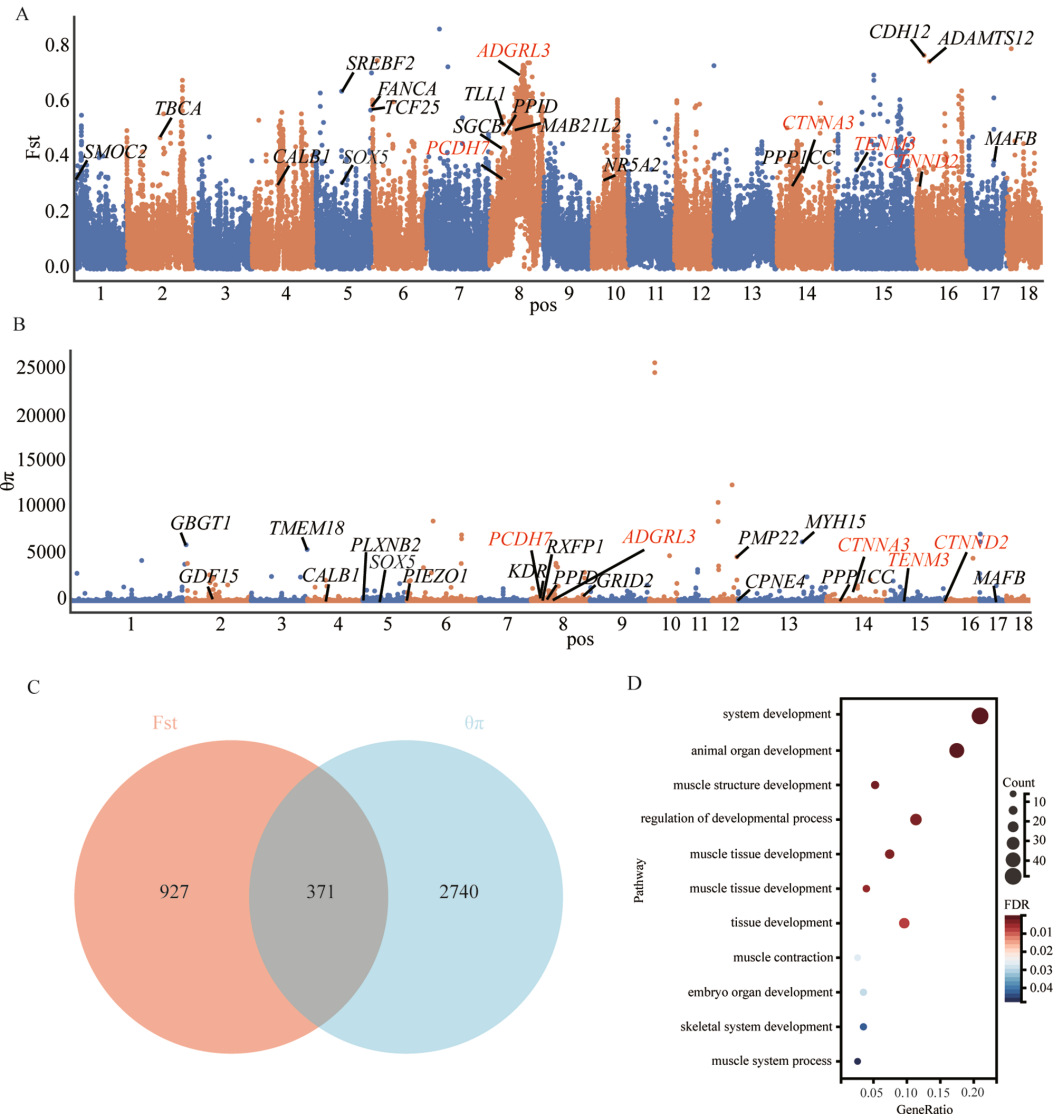
Genome-wide distribution of selection signatures detected by Fst, θπ on 18 chromosomes from top to bottom. (**A**) Represents the Fst. (**B**) represents the θπ between QCB pigs and LW pigs. (**C**) Represent the gene number distribution of candidate signal intervals between QCB pigs and LW pigs populations screened by two methods. (**D**) Represents GO enrichment analysis of some significantly enriched genes in the selected region of QCB pigs

To identify gene loci that have undergone strong selection signatures during the domestication process shared by both QCB Pigs and LW Pigs. Two methods for genomic selection signature detection, including population differentiation coefficient (Fst) and polymorphism levels statistic (θπ) were performed to detect the genomic regions under selection in QCB pigs by comparing with LW. In this study, only a region that was within the top 5% of the Fst and θπ ratio could be identified as a selected region. A total of 7331 regions were identified in the top 5% of the two statistics. The Manhattan plot of the two statistics across autosomes is shown in Fig. 3A, B. After combining the two statistics, 371 genes were identified in the selected regions (Fig. 3C). To assess the function of the positive selection genes (PSGs), GO terms were determined using g: profiler with a corrected *P*-value of less than 0.05 as significant. 371 positive selection genes (PSGs) under selection were utilized for the GO analysis. The results revealed a significant enrichment of GO pathways such as muscle tissue development (*EDNRA*, *SGCD*), system development (*SOX5*, *CTNND2*, *TENM3*), and tissue development (*EDNRA*, *SOX5*, *MAFB*) etc. (Fig. 3D).

### SNPs detected by single-locus GWAS

**Fig. 4:**
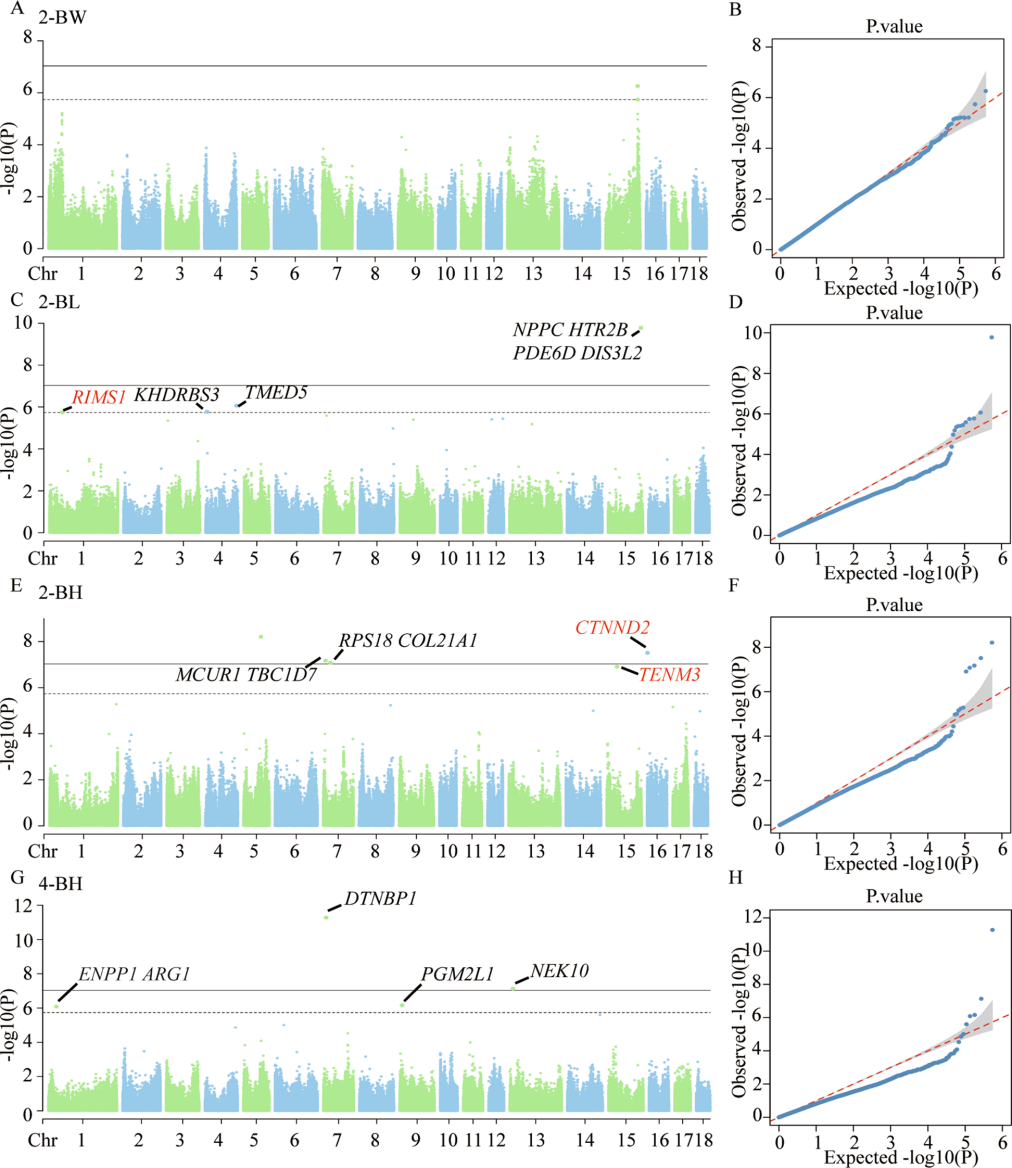
Manhattan and Q-Q plots of single-Locus GWAS based on imputation using SWIM reference panel for four growth traits. (**A, C, E, G**) Manhattan plots for 2-BW, 2-BL, 2-BH, 4-BH. (**B, D, F, H**) Quantitative-Quantitative (Q-Q) plots for 2-BW, 2-BL, 2-BH, 4-BH. The solid line represents the genome-wide significance level (9.25 × 10^− 8^); The dashed line represents the suggestive significance. Abbreviations: 2-BW = body weight at 2 months, 2-BL = body length at 2 months, 2-BH = body height at 2 months, and 4-BH = body height at 4 months

Phenotype data on growth traits of 197 were selected of 460 QCB pigs, The 197 low-depth whole-genome resequencing data were imputed to resequencing data using SWIM reference panel imputation databases. Figure 4 shows the results of Manhattan plots and Q-Q plots after imputation using SWIM reference panel imputation databases. GWAS was performed on 197 individuals using the FarmCPU model to identify candidate genes for growth traits in QCB pigs. The results show that 1, 4 and 2 genome-wide significant SNPs [*P* < 9.25 × 10^− 8^(0.05/540,759)] for 2-BL, 2-BH and 4-BH are identified, respectively (Table 2). Notably, 11 genes are identified as related to growth, including *NPPC*, *HTR2B*, *PDE6D*, *DIS3L2*, *MCUR1*, *TBC1D7*, *RPS18*, *COL21A1*, *CTNND2*, *DTNBP1* and *NEK10*. According to the suggestive significance threshold [*P* < 1.85 × 10^− 6^(1/540,759)], 2, 3, 1, and 2 SNPs were found to be associated with 2-BW, 2-BL, 2-BH, and 4-BH, respectively (Table 3). And 7 genes were identified as related to growth, including *RIMS*, *KHDRBS3*, *TMED5*, *TENM3*, *ENPP1*, *ARG1* and *PGM2L1*. While there were no genome-wide significant SNPs for the six growth traits including 2-CC, 2-TC, 4-BW, 4-BL, 4-CC, 4-TC (Fig. S2). In this study, candidate genes were found by searching 0.5 Mb upstream and downstream of the significant and suggestive significant SNPs using GWAS based on imputed databases (Table S2).

**Table 2:** Genome-wide significant SNPs for growth traits

Traits	SNP^1^	Chr^2^	Position	*P*- Value	Candidate Gene
2-BL^3^	15: 132,315,138	15	132,315,138	1.67E-10	*NPPC HTR2B PDE6D DIS3L2*
2-BH^4^	5: 70,115,422	5	70,115,422	6.16E-09	*-*
2-BH	7: 9,879,713	7	9,879,713	6.70E-08	*MCUR1 TBC1D7*
2-BH	7: 29,282,528	7	29,282,528	8.35E-08	*RPS18 COL21A1*
2-BH	16: 1,154,491	16	1,154,491	3.07E-08	*CTNND2*
4-BH^5^	7: 11,211,250	7	11,211,250	5.28E-12	*DTNBP1*
4-BH	13: 13,335,332	13	13,335,332	7.36E-08	*NEK10*

^1^SNP: single-nucleotide polymorphism; ^2^Chr: chromosome; ^3^2-BL: body length at 2 months; ^4^2-BH: body height at 2 months; ^5^4-BH: body height at 4 months

**Table 3:** Genome-wide suggestive significant SNPs for growth traits

Traits	SNP^1^	Chr^2^	Position	*P*- Value	Candidate Gene
2-BW^3^	15: 129,809,430	15	129,809,430	1.82E-06	*-*
2-BW	15: 129,888,533	15	129,888,533	5.48E-07	*-*
2-BL^4^	1: 52,055,241	1	52,055,241	1.79E-06	*RIMS1*
2-BL	4: 5,817,122	4	5,817,122	1.69E-06	*KHDRBS3*
2-BL	4: 124,246,502	4	124,246,502	8.59E-07	*TMED5*
2-BH^5^	15: 41,506,661	15	41,506,661	1.24E-07	*TENM3*
4-BH^6^	1: 31,679,073	1	31,679,073	8.22E-07	*ENPP1 ARG1*
4-BH	9: 8,996,518	9	8,996,518	6.94E-07	*PGM2L1*

^1^SNP: single-nucleotide polymorphism; ^2^Chr: chromosome; ^3^2-BW: body wength at 2 months; ^4^2-BL: body length at 2 months; ^5^2-BH: body height at 2 months; ^6^4-BH: body height at 4 months

### SNPs detected by multi-locus GWAS

**Fig. 5:**
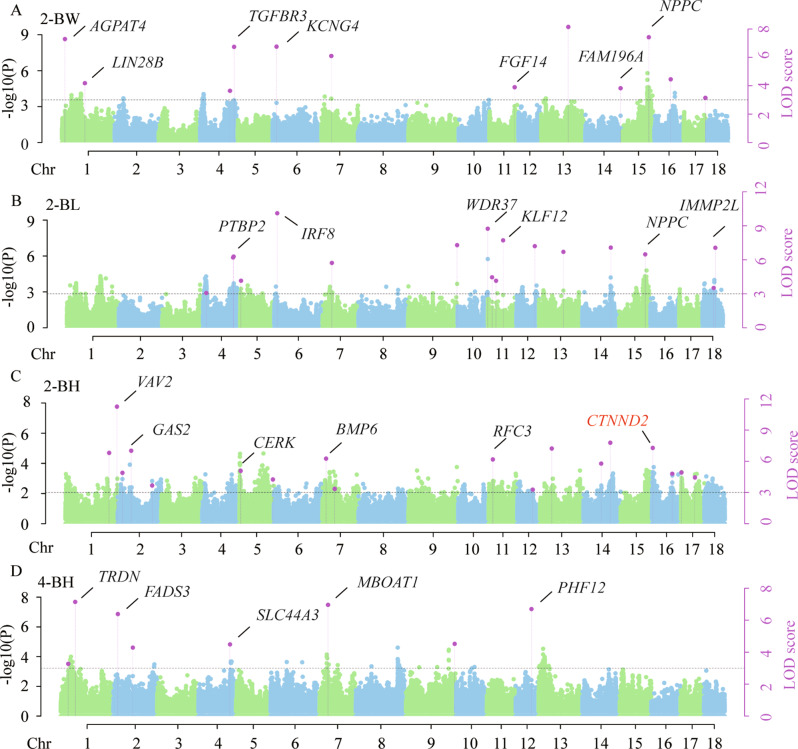
Manhattan plots of multi-Locus GWAS based on imputation using SWIM reference panel for four growth traits. (**A, B, C, D**) Manhattan plots for 2-BW, 2-BL, 2-BH, 4-BH. Manhattan plots indicate LOD scores for genome-wide SNPs (y-axis) plotted against their respective positions on each chromosome (x-axis), and the horizontal lines indicate the thresholds for significance (LOD score = 3). Abbreviations: 2-BW = body weight at 2 months, 2-BL = body length at 2 months, 2-BH = body height at 2 months, and 4-BH = body height at 4 months

A total of 123 significant SNPs (LOD score > 3) were detected (Table 4; Fig. 5, Fig. S3, Table S3) by multi-locus method, and the results show that 12, 17, 18, 18, 15, 12, 7, 8, 5 and 11 significant SNPs (LOD score > 3) for 2-BW, 2-BL, 2-BH 2-CC, 2-TC, 4-BW, 4-BL, 4-BH, 4-CC, and 4-TC are identified, respectively. Interestingly, the SNP rs1154491 located on SSC 16 was detected by all two methods. And *CTNND2* gene was all identified as related to 2-BH.

**Table 4:** Description of significant SNPs identified by multi-locus method as associated with growth traits

Traits	SNP^1^	Chr^2^	Position	LOD score	*r*^2^(%)^3^	Candidate Gene
2-BW^4^	1:6396933	1	6,396,933	7.29	4.25	*AGPAT4*
2-BW	1:71445572	1	71,445,572	4.18	2.01	*LIN28B*
2-BW	4:119264884	4	119,264,884	3.64	1.10	*SNX7*
2-BW	4:124914137	4	124,914,137	6.74	5.41	*TGFBR3*
2-BW	6:4331800	6	4,331,800	6.76	5.45	*KCNG4*
2-BW	7:19094139	7	19,094,139	6.10	5.22	*KIAA0319*
2-BW	11:70447343	11	70,447,343	3.90	1.26	*FGF14*
2-BW	13:103784734	13	103,784,734	8.14	4.70	*-*
2-BW	14:136754144	14	136,754,144	3.83	2.43	*FAM196A*
2-BW	15:132340482	15	132,340,482	7.42	6.18	*NPPC*
2-BW	16:29188289	16	29,188,289	4.46	3.31	*FGF10*
2-BW	17:59259520	17	59,259,520	3.15	1.67	*CTSZ*
2-BL^5^	4:7396038	4	7,396,038	3.07	1.06	*ZFAT*
2-BL	4:120486137	4	120,486,137	6.20	2.74	*PTBP2*
2-BL	4:121538115	4	121,538,115	6.29	2.39	*PTBP2*
2-BL	5:2278258	5	2,278,258	4.15	1.13	*TBC1D22A*
2-BL	6:3535262	6	3,535,262	10.11	5.91	*IRF8*
2-BL	7:17568079	7	17,568,079	5.72	2.15	*PRL*
2-BL	9:138483163	9	138,483,163	7.30	3.29	*-*
2-BL	10:68503232	10	68,503,232	8.75	4.22	*WDR37*
2-BL	11:10434854	11	10,434,854	4.46	1.34	*RFC3*
2-BL	11:19433095	11	19,433,095	4.15	1.45	*MED4*
2-BL	11:46552378	11	46,552,378	7.72	2.79	*KLF12*
2-BL	12:54143906	12	54,143,906	7.20	2.88	*USP43*
2-BL	13:85852217	13	85,852,217	6.70	2.85	*-*
2-BL	14:105024482	14	105,024,482	7.08	2.79	*SLC35G1*
2-BL	15:132873410	15	132,873,410	6.48	2.74	*NPPC*
2-BL	18:31038583	18	31,038,583	3.51	1.16	*FOXP2*
2-BL	18:35051931	18	35,051,931	7.06	3.34	*IMMP2L*
2-BH^6^	1:250878677	1	250,878,677	6.81	2.67	*PTPN3*
2-BH	1:273386165	1	273,386,165	11.26	4.79	*VAV2*
2-BH	2:10667280	2	10,667,280	4.89	1.88	*DDB1*
2-BH	2:36547982	2	36,547,982	7.01	1.82	*GAS2*
2-BH	2:132847827	2	132,847,827	3.67	1.22	*CHSY3*
2-BH	5:2545703	5	2,545,703	5.08	2.25	*CERK*
2-BH	5:99882722	5	99,882,722	4.26	1.79	*ACSS3*
2-BH	7:5179968	7	5,179,968	6.26	2.32	*BMP6*
2-BH	7:24919117	7	24,919,117	3.35	1.43	*BMP5*
2-BH	11:10434854	11	10,434,854	6.18	1.71	*RFC3*
2-BH	12:44345838	12	44,345,838	3.28	1.54	*FOXN1*
2-BH	13:25791420	13	25,791,420	7.23	3.53	*KLHL40*
2-BH	14:54246154	14	54,246,154	5.78	2.69	*ACTN2*
2-BH	14:96198734	14	96,198,734	7.78	4.18	*PCDH15*
2-BH	16:797534	16	797,534	7.28	4.40	*CTNND2*
2-BH	16:54503683	16	54,503,683	4.80	1.31	*SLIT3*
2-BH	17:811086	17	811,086	4.92	2.33	*PRAG1*
2-BH	17:42516390	17	42,516,390	4.44	1.99	*DHX35*
4-BH^7^	1:19829505	1	19,829,505	3.27	1.73	*FBXO30*
4-BH	1:38953464	1	38,953,464	7.15	6.34	*TRDN*
4-BH	2:9782979	2	9,782,979	6.38	6.14	*FADS3*
4-BH	2:72348683	2	72,348,683	4.29	3.15	*INSR*
4-BH	4:122434064	4	122,434,064	4.49	7.02	*SLC44A3*
4-BH	7:15360062	7	15,360,062	6.96	6.86	*MBOAT1*
4-BH	9:136718449	9	136,718,449	4.52	6.49	*GRB10*
4-BH	12:45097165	12	45,097,165	6.70	5.65	*PHF12*

^1^SNP: single-nucleotide polymorphism; ^2^Chr: chromosome; ^3^2-BW: body weight at 2 months; ^4^2-BL: body length at 2 months; ^5^2-BH: body height at 2 months; ^6^4-BH: body height at 4 months

### Differential expressed gene analysis

**Fig. 6:**
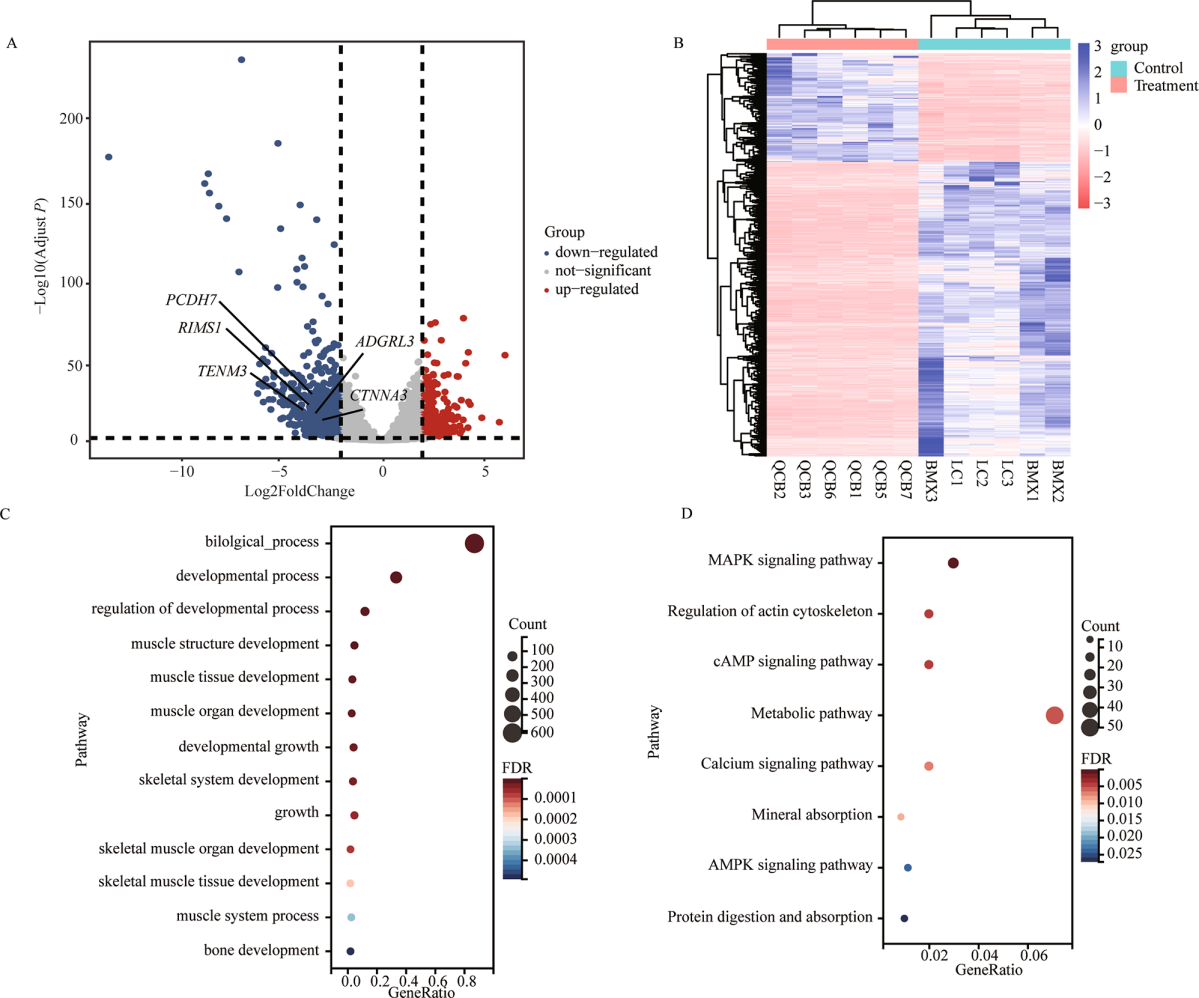
Differentially expressed genes (DEGs) between Control and Treatment. (**A**) Control vs. Treatment volcano plot. (**B**) Control vs. Treatment heat map. (**C**) Kyoto encyclopedia of genes and genomes (KEGG) enrichment analysis of the differentially expressed genes (DEGs) between the Control vs. Treatment. (**D**) (KEGG) enrichment analysis of the differentially expressed genes (DEGs) between the Control vs. Treatment

A total of 14,541 genes were obtained from RNA-seq analysis (Fig. 6A). After quality control, we found 1137 DEGs between control and treatment, of which 307 genes were up-regulated and 830 genes were down-regulated (Fig. 6A). A clear distinction between control and treatment comparisons was shown by clustering analysis according to the DEGs (Fig. 6B). For the 1137 DEGs between control and treatment, the genes were mapped to GO and KEGG. The significant biological process (*P* < 0.05) that may be involved in growth were the muscle tissue development, muscle organ development, development growth etc. (Fig. 6C, Table S4). The significant pathways (*P* < 0.05) that may be involved in growth were the MAPK, cAMP, Calcium, AMPK pathways etc. (Fig. 6D, Table S5).

### Screening of candidate genes

**Fig. 7:**
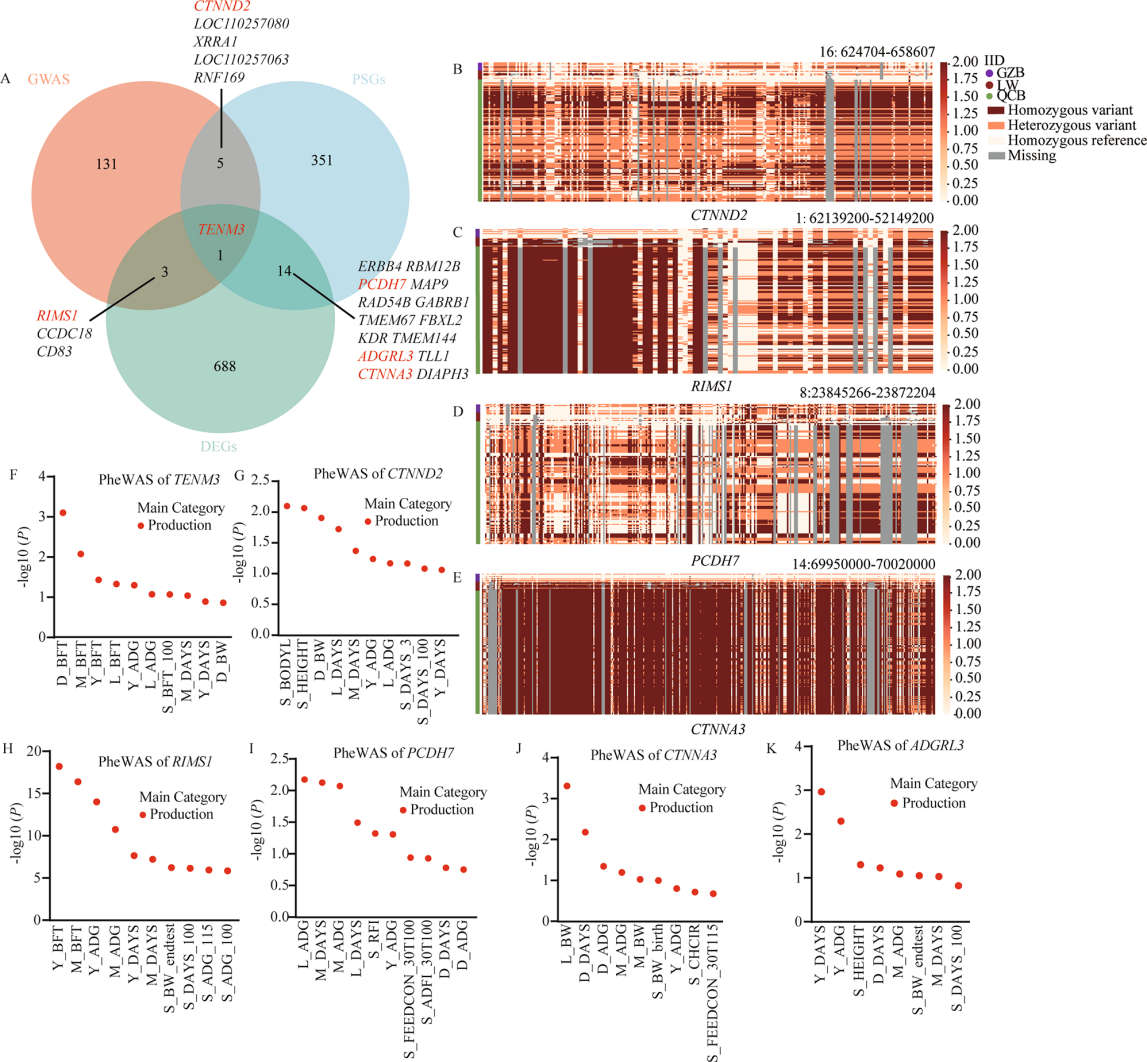
Identification of the candidate genes. (**A**) Venn diagram of three methods to identify candidate genes. (**B**) The genotype of the *CTNND2* gene. (**C**) The genotype of the *RIMS1* gene. (**D**) The genotype of the *PCDH7* gene. (**E**) The genotype of the *CTNNA3* gene. (**F**) Phenome-wide association (PheWAS) analysis of *TENM3* with the top 10 production traits. (**G**) Phenome-wide association (PheWAS) analysis of *CTNND2* with the top 10 production traits. (**H**) Phenome-wide association (PheWAS) analysis of *RIMS1 *with the top 10 production traits. (**I**) Phenome-wide association (PheWAS) analysis of *PCDH7* with the top 10 production traits. (**J**) Phenome-wide association (PheWAS) analysis of *CTNNA3* with the top 9 production traits. (**K**) Phenome-wide association (PheWAS) analysis of *ADGRL3* with the top 8 production traits

Among the candidate genes identified by selection signatures, GWAS and transcriptome analyses (Fig. 7A), the *TENM3* gene were overlapped between the candidate gene from GWAS, PSGs and DEGs (Fig. S4A), the *CTNNA2* gene was overlapped between the candidate gene from GWAS and PSGs, the *RIMS1* gene was overlapped between the candidate gene from GWAS and DEGs (Fig. 7B, C), and the *PCDH7*, *CTNNA3* and gene were overlapped between the candidate gene from PEGs and DEGs (Fig. 7D, E, Fig. S4B). Genotype analysis revealed that the genotype at the gene loci of some QCB pigs were consistent with those of LW pigs and GZB pigs. The PigBiobank analyses further validated that *TENM3*, *CTNNA2*, *RIMS1*, *PCDH7*, *ADGRL3* and *CTNNA3* gene are associated with growth traits (Fig. 7F-K).

## Discussion

Growth traits such as BW, BL, BH, CC, and TC are closely related to pig growth and are important quantitative indicators of pig growth. It has been reported that the heritability of growth traits is in the range of 0.13-0.73 [34, 35], which indicates a medium heritability. This is consistent with the results of our study, in which the heritability of growth parameters (BW, BL, TC) ranged from 0.2390 to 0.5049, However, the heritability of 4-BH and 4-CC was different. In our study, the heritability of 4-BH and 4-CC was 0.0316 and 0.0293, this might be due to its growth not being stable during the breeding process in this study. In addition, genetic correlations between individual traits were analyzed, demonstrating significant positive relationships among BW, BL, BH, CC, and TC at two and four months. Except for 4-BL and 4-BH, 4-BH and 4-CC and 4-CC and 4-TC with negative correlation, the correlation of other growth parameters ranging from 0.1507 to 0.9208, this might be due to the external environmental factors and human factors have a certain impact on the growth and development of QCB pigs. This suggests that fewer traits can be selected to simplify breeding work.

With the rapid development of sequencing technologies, reduced costs, and increasing demand for high-density markers, genotype imputation has become a widely utilized tool in recent years. Its accuracy depends on the density of target SNPs, the platforms used for imputation, and the software employed [36]. Nowadays, there are increasing numbers of large reference panels used for effective phasing and imputation of whole-genome microarray chips and low-coverage sequencing data, such as the Pig Haplotype Reference Panel (PHARP) database and the Swine Reference [18] Haplotype Panel (SWIM) [17, 37]. Moreover, various genotype imputation software options are available, such as Beagle [38], PLINK [22], IMPUTE2 [9], Shapeit4 [39], STITCH [18] and QUILT [40]. Teng et al. [14] leveraging a multibreed pig genomics reference panel (PGRP) consisting of 1,602 WGS samples, imputed genotypes of samples with an imputation accuracy of 94% (concordance rate) and 0.82 (genotype correlation, r^2^). In this study, we leveraged a reference panel of SWIM, imputed genotypes of samples with an imputation accuracy of 95.83% (concordance rate) and 0.86 (genotype correlation, r^2^), prove that our results are reliable. Additionally, there are no resequencing individuals of QCB pigs and GZB pigs in the SWIM reference panel, there is still improvement in imputation accuracy.

Genetic structure within populations undergo changes during the breeding process, influenced by factors like natural selection and artificial breeding [41]. This result in a loss of genetic diversity within the population, highlighting the importance of researching population genetic structure using molecular marker techniques [42]. To better understand the characteristics of the germplasm in QCB pigs, we sequenced 197 unrelated QCB pigs and obtained the whole genome variations. Afterward, a total of 44 pig breeds (410 samples) were combined to analyze the population structure. Through NJ tree, PCA, and Admixture showed distinct lineages between Asian pigs and European pigs. And the QCB pigs were closer to the LW pigs. This is likely due to QCB pigs being in the early stages of breeding, with their genetic lineage and traits still evolving. LD analysis indicated that QCB pigs display a greater level of genetic diversity similar to other local breeds, such as hybrids sheep [41], Licha black pigs [43], and Beijing black pigs [44].

Selection signatures help to identify the genes that natural selection has shaped and molded adaptive traits in different species, thus improving the understanding of biological evolution and adaptation. Whereas different detection methods may have different results, we usually use multiple methods to eliminate the probability of generating false positives, so as to detect regions under selection and screen for candidate genes associated with their traits. In this study, we used Fst and θπ two methods. Fst, which is based on population differentiation, measures the degree of differentiation between populations by analyzing allele frequencies of individual SNPs [45-47]. θπ, based on nucleotide diversity, estimates population differentiation by assessing the level of nucleotide diversity. Previous studies by Pan et al. [48] and Xu et al. [49] utilized similar methods in small pig breeds and sheep breeds, respectively, to identify candidate genes associated with various phenotype such as pig conformation, milk production, ear size, and body conformation. To investigate the candidate genes affecting the growth traits of QCB pigs, a total of 371 PSGs were identified in the selected regions, respectively. Functional enrichment analysis revealed that these genes were related to muscle tissue development, tissue development and system development.

The sequencing data of 197 QCB pigs were analyzed using the FarmCPU model for GWAS of 10 growth traits. The FarmCPU model has a faster computational speed, while being able to fully utilize the population genome information to reduce errors and improve accuracy. A total of 18 candidate genes were identified for the growth traits. Unfortunately, no potential SNP was identified for the other six growth traits, probably due to the small size of the population and the high number of missing phenotype data points. Despite its simplicity and speed, single-locus analysis makes a strong assumption that only one QTL has an effect. This is largely valid for polygenic traits, where QTLs other than the one being tested can be properly accounted for by the polygenic term. In this study, we used both the single-locus analysis and multi-locus analysis to overcome some of the limitations in single-locus analysis. Standard multi-locus GWAS has two stages. In the first stage, a candidate subset of markers is selected through single-locus MLM. After this stage, putative markers are added to the model iteratively until a certain selection criterion is met [50]. Such multi-locus model can reduce bias in the effect estimates and improve power to detect associations [51, 52]. By combining both single-locus and multi-locus methods, we all found *CTNND2* candidate gene that associated with growth trait. In general, our study demonstrated that improved efficiency and accuracy could be achieved by a combination of the single-locus and multi-locus GWAS for identification of growth-related QTLs in QCB pigs.

In recent years, the use of transcriptomic to identify differentially expressed genes affecting economically important traits has also become a mainstream approach. The longissimus dorsi muscle is closely related to the growth and development of animals. We collected QCB pig and small pig longissimus dorsi muscle s. To further explore the genes related to growth traits, a total of 1137 DEGs were identified by differentially expressed gene analysis of RNA-seq data from control and treatment groups, including 307 up-regulated genes and 830 down-regulated genes. A total of 18 DEGs were involved in the significantly enriched GO and KEGG pathways closely related to muscle and growth and development, in which five genes were overlapped between the candidate genes from GWAS, PSGs and DEGs: *TENM3*, *RIMS1*, *PCDH7*, *ADGRL3* and *CTNNA3*.

The genome and transcriptome technologies could analyze the molecular mechanism of complex traits in pigs, which have advantages such as high resolution, good quantitation, and deep coverage. Liu et al. [53] integrative analysis of GWAS loci, eQTL and QTL demonstrated *GALNT15*/*GALNTL2* and *HTATIP2* as strong candidate genes for drip loss and pH drop from postmortem 45 min to 24 h, respectively. Ibragimov et al. [54] search for quantitative trait loci (QTL), candidate genes, and biological pathways associated with FE using both genotype and RNA-seq data. However, less studies have been conducted to analyze the growth of pigs with genome and transcriptome data. In our study, these PSGs, GWAS and DEGs (*TENM3*, *CTNND2*, *RIMS1*, *PCDH7*, *ADGRL3* and *CTNNA3*) may affect the growth traits in QCB pigs. *TENM3* gene encodes highly conserved type II transmembrane glycoprotein, and it has been proposed that mutations in this gene cause a slowed chondrogenesis and slow growth [55].*CTNND2* gene belongs to the actin alpha interferon/intrinsic protein alpha interferon family, and this gene has been studied in pig body size and litter weight, which plays a key role in growth, development, and energy metabolism [5, 56]. *RIMS1* is a member of the Ras gene superfamily and autosomal dominant conosone dystrophy 7 (*CORD7*) was originally associated with the *RIMS1* gene [57]. *PCDH7* belongs to the protocadherin gene family, and was performed for growth traits at each growth stage in Simmental beef cattle [58]. *ADGRL3* is a member of the latrophilin subfamily that encodes G protein-coupled receptors (GPCRs), the gene was involved in cell differentiation and energy metabolism, which associated with growth, efficiency and carcass traits in Sant-Ynez sheep [59]. *CTNNA3* is a key protein in epithelial cell adhesion junction complexes, and *CTNNA3* mutation (g.2018018 A > G) was significantly associated with weight, height, body length and chest circumference in Hu sheep [60].

The primary limitation of this study lies in the breeding population is still in its early developmental stages. We plan to continue data collection in the future. To enhance the reliability of our findings, we employed multiple approaches to jointly identify candidate genes associated with growth traits.

## Conclusion

In this study, population structure, selection signatures and GWAS of QCB pigs were analyzed using the imputed low-depth whole-genome resequencing data for the first time. A total of six candidate genes (*TENM3*, *CTNND2*, *RIMS1*, *PCDH7*, *ADGRL3* and *CTNNA3*) related to growth traits were identified in this study. Our findings will improve breeding, analyze the genetic basis of growth traits and provide a theoretical basis for accelerating the breeding of QCB pigs.

## Electronic supplementary material

Below is the link to the electronic supplementary material.

Supplementary Material 1

## Data Availability

The data that support the findings of this study have been deposited into Genome Sequence Archive (GSA) of China National Center Bioinformation (CNCB) with accession number CRA023463 (Browse-BioProject-CNCB-NGDC).

## Abbreviations

GEO: Gene expression omnibus data base
BWA: Burrows-wheeler aligner
VCF: Variant call formats
MAF: Minorganic allele frequencies
HWE: Hardy-weinberg equilibrium
QCB: Qinchuan black
GZB: Guanzhong black
LW: Large white
NJ: Neighbor-joining
PCA: Principal component analysis
LD: Linkage disequilibrium
Fst: F-statistics
θπ: Nucleotide diversity
GO: Gene ontology
KEGG: Kyoto encyclopedia of genes and genomes
BW: Body weight
BL: Body length
CC: Chest circumference
BH: Body height
TC: Tube circumference
GAPIT: Genome association and prediction integrated tool
FarmCPU: Fixed and random model circulating probability unification
GWAS: Genome-wide association study
SNP: Single nucleotide polymorphism
DEG: Differential expression gene
PSG: Positive selection gene
