# Supplementary material for: Whole genome and transcriptome analyses identify genetic markers associated with growth traits in Qinchuan black pig
Source: BMC Genomics. 2025 May 12;26:469. doi: 10.1186/s12864-025-11627-5 (PMC12067757; doi:10.1186/s12864-025-11627-5)
Supplement: Supplementary file 1 — Supplementary Material 1 [file 12864_2025_11627_MOESM1_ESM.docx]

**Table S1** Public database download information

| Project Number | Sample Number | Breed | Sequencing Technology | Depth |
| --- | --- | --- | --- | --- |
| [PRJEB1683](https://www.ncbi.nlm.nih.gov/bioproject/PRJEB1683) | SAMEA1557418 | Warthog | Illumina HiSeq 2000 | 12.39 |
| PRJEB1683 | SAMEA1557396 | Wild Bor South China | Illumina HiSeq 2000 | 11.03 |
| PRJEB1683 | SAMEA1557421 | Wild Boar North China | Illumina HiSeq 2000 | 10.89 |
| PRJEB1683 | SAMEA2612519 | Asian wild | Illumina HiSeq 2000 | 9.58 |
| PRJEB1683 | SAMEA2612520 | Asian wild | Illumina HiSeq 2000 | 13.02 |
| PRJEB1683 | SAMEA2612521 | Asian wild | Illumina HiSeq 2000 | 11.68 |
| PRJNA213179 | SAMN02298086 | BGI_CWB_ZJWB02 | Illumina HiSeq 2000 | 19.31 |
| PRJNA213179 | SAMN02298085 | BGI_CWB_ZJWB01 | Illumina HiSeq 2000 | 18.35 |
| PRJNA213179 | SAMN02298084 | BGI_CWB_SYWB205 | Illumina HiSeq 2000 | 19.04 |
| PRJNA213179 | SAMN02298083 | BGI_CWB_SYWB103 | Illumina HiSeq 2000 | 23.47 |
| PRJNA213179 | SAMN02298082 | BGI_CWB_NCYZ0013 | Illumina HiSeq 2000 | 24.70 |
| PRJNA213179 | SAMN02298081 | BGI_CWB_NCYZ0010 | Illumina HiSeq 2000 | 23.45 |
| PRJEB9922 | SAMEA3497816 | ASW_SChina | Illumina HiSeq 2000 | 11.31 |
| PRJEB9922 | SAMEA3497818 | ASW_SChina | Illumina HiSeq 2000 | 28.62 |
| PRJEB9922 | SAMEA3497819 | ASW_SChina | Illumina HiSeq 2000 | 13.98 |
| PRJEB9922 | SAMEA3497821 | ASW_SChina | Illumina HiSeq 2000 | 11.10 |
| PRJEB9922 | SAMEA3497822 | ASW_SChina | Illumina HiSeq 2000 | 13.09 |
| PRJEB1683 | SAMEA2612513 | European wild | Illumina HiSeq 2000 | 11.06 |
| PRJEB1683 | SAMEA2612514 | European wild | Illumina HiSeq 2000 | 12.94 |
| PRJEB1683 | SAMEA2612515 | European wild | Illumina HiSeq 2000 | 14.55 |
| PRJEB1683 | SAMEA2612516 | European wild | Illumina HiSeq 2000 | 10.00 |
| PRJEB1683 | SAMEA2612517 | European wild | Illumina HiSeq 2000 | 9.58 |
| PRJEB1683 | SAMEA2612518 | European wild | Illumina HiSeq 2000 | 12.42 |
| PRJEB1683 | SAMEA2612522 | European wild | Illumina HiSeq 2000 | 11.93 |
| PRJEB1683 | SAMEA2612523 | European wild | Illumina HiSeq 2000 | 10.88 |
| PRJEB1683 | SAMEA2612524 | European wild | Illumina HiSeq 2000 | 13.05 |
| PRJEB1683 | SAMEA2612525 | European wild | Illumina HiSeq 2000 | 12.70 |
| PRJEB1683 | SAMEA2612526 | European wild | Illumina HiSeq 2000 | 11.68 |
| PRJEB9922 | SAMEA3497864 | EUW_Netherlands | Illumina HiSeq 2000 | 11.90 |
| PRJEB9922 | SAMEA3497866 | EUW_Netherlands | Illumina HiSeq 2000 | 10.69 |
| PRJEB9922 | SAMEA3497867 | EUW_Netherlands | Illumina HiSeq 2000 | 13.32 |
| PRJEB9922 | SAMEA3497868 | EUW_Netherlands | Illumina HiSeq 2000 | 11.89 |
| PRJEB9922 | SAMEA3497869 | EUW_Netherlands | Illumina HiSeq 2000 | 16.53 |
| PRJEB9922 | SAMEA3497870 | EUW_Netherlands | Illumina HiSeq 2000 | 8.25 |
| PRJEB9922 | SAMEA3497871 | EUW_Netherlands | Illumina HiSeq 2000 | 9.15 |
| PRJEB9922 | SAMEA3497872 | EUW_Netherlands | Illumina HiSeq 2000 | 10.69 |
| PRJEB9922 | SAMEA3497873 | EUW_Netherlands | Illumina HiSeq 2000 | 10.22 |
| PRJEB9922 | SAMEA3497874 | EUW_Netherlands | Illumina HiSeq 2000 | 14.53 |
| PRJEB9922 | SAMEA3497875 | EUW_Netherlands | Illumina HiSeq 2000 | 13.26 |
| PRJEB9922 | SAMEA3497876 | EUW_Netherlands | Illumina HiSeq 2000 | 11.14 |
| PRJEB9922 | SAMEA3497877 | EUW_Netherlands | Illumina HiSeq 2000 | 9.84 |
| PRJEB9922 | SAMEA3497878 | EUW_Netherlands | Illumina HiSeq 2000 | 15.28 |
| PRJEB9922 | SAMEA3497879 | EUW_Netherlands | Illumina HiSeq 2000 | 13.01 |
| PRJEB9922 | SAMEA3497880 | EUW_Netherlands | Illumina HiSeq 2000 | 7.45 |
| PRJEB1683 | SAMEA2612511 | Asian domestic | Illumina HiSeq 2000 | 11.27 |
| PRJEB1683 | SAMEA2612512 | Asian domestic | Illumina HiSeq 2000 | 9.73 |
| PRJEB1683 | SAMEA2628062 | Asian domestic | Illumina HiSeq 2000 | 11.15 |
| PRJEB1683 | SAMEA2628063 | Asian domestic | Illumina HiSeq 2000 | 11.05 |
| PRJNA186497 | SAMN01894360 | Tibetan pig | Illumina HiSeq 2000 | 6.18 |
| PRJNA186497 | SAMN01894349 | Tibetan pig | Illumina HiSeq 2000 | 4.88 |
| PRJNA186497 | SAMN01894345 | Tibetan pig | Illumina HiSeq 2000 | 5.42 |
| PRJNA186497 | SAMN01894457 | Jinhua pig | Illumina HiSeq 2000 | 5.02 |
| PRJNA186497 | SAMN01894456 | Jinhua pig | Illumina HiSeq 2000 | 5.87 |
| PRJNA186497 | SAMN01894455 | Jinhua pig | Illumina HiSeq 2000 | 5.39 |
| PRJNA186497 | SAMN01894452 | Neijiang pig | Illumina HiSeq 2000 | 5.30 |
| PRJNA186497 | SAMN01894448 | Neijiang pig | Illumina HiSeq 2000 | 7.22 |
| PRJNA186497 | SAMN01894447 | Neijiang pig | Illumina HiSeq 2000 | 5.96 |
| PRJNA186497 | SAMN01894446 | Ya'nan pig | Illumina HiSeq 2000 | 6.02 |
| PRJNA186497 | SAMN01894445 | Ya'nan pig | Illumina HiSeq 2000 | 5.14 |
| PRJNA186497 | SAMN01894444 | Ya'nan pig | Illumina HiSeq 2000 | 5.49 |
| PRJNA186497 | SAMN01894442 | Wujin pig | Illumina HiSeq 2000 | 6.23 |
| PRJNA186497 | SAMN01894441 | Wujin pig | Illumina HiSeq 2000 | 6.57 |
| PRJNA213179 | SAMN02298141 | BGI_EHL_0F0094 | Illumina HiSeq 2000 | 24.19 |
| PRJNA213179 | SAMN02298140 | BGI_EHL_0F0090 | Illumina HiSeq 2000 | 24.18 |
| PRJNA213179 | SAMN02298139 | BGI_EHL_0F0202 | Illumina HiSeq 2000 | 23.20 |
| PRJNA213179 | SAMN02298080 | BGI_EHL_ER_CS3544 | Illumina HiSeq 2000 | 23.89 |
| PRJNA213179 | SAMN02298079 | BGI_EHL_ER_CS0234 | Illumina HiSeq 2000 | 24.16 |
| PRJNA213179 | SAMN02298138 | BGI_LWH_LWH1183 | Illumina HiSeq 2000 | 23.21 |
| PRJNA213179 | SAMN02298137 | BGI_LWH_LWH879 | Illumina HiSeq 2000 | 23.28 |
| PRJNA213179 | SAMN02298136 | BGI_LWH_LWH181 | Illumina HiSeq 2000 | 23.70 |
| PRJNA213179 | SAMN02298135 | BGI_LWH_LWH13 | Illumina HiSeq 2000 | 24.53 |
| PRJNA213179 | SAMN02298134 | BGI_LWH_LWH0M | Illumina HiSeq 2000 | 21.72 |
| PRJNA213179 | SAMN02298133 | BGI_LWH_LWH0F | Illumina HiSeq 2000 | 23.00 |
| PRJNA213179 | SAMN02298126 | BGI_MIN_MZhu3126 | Illumina HiSeq 2000 | 23.01 |
| PRJNA213179 | SAMN02298125 | BGI_MIN_MZhu3312 | Illumina HiSeq 2000 | 23.86 |
| PRJNA213179 | SAMN02298124 | BGI_MIN_MZhu3252 | Illumina HiSeq 2000 | 21.03 |
| PRJNA213179 | SAMN02298123 | BGI_MIN_MZhu3134 | Illumina HiSeq 2000 | 22.92 |
| PRJNA213179 | SAMN02298122 | BGI_MIN_MZ-307-00 | Illumina HiSeq 2000 | 22.60 |
| PRJNA213179 | SAMN02298121 | BGI_MIN_MZ-304-07 | Illumina HiSeq 2000 | 22.80 |
| PRJNA213179 | SAMN02298132 | BGI_BMX_BMX0015 | Illumina HiSeq 2000 | 24.22 |
| PRJNA213179 | SAMN02298131 | BGI_BMX_BMX0014 | Illumina HiSeq 2000 | 23.99 |
| PRJNA213179 | SAMN02298130 | BGI_BMX_BMX0013 | Illumina HiSeq 2000 | 24.09 |
| PRJNA213179 | SAMN02298129 | BGI_BMX_BMX0012 | Illumina HiSeq 2000 | 23.36 |
| PRJNA213179 | SAMN02298128 | BGI_BMX_BMX0011 | Illumina HiSeq 2000 | 25.02 |
| PRJNA213179 | SAMN02298127 | BGI_BMX_BMX0010 | Illumina HiSeq 2000 | 23.65 |
| PRJNA213179 | SAMN02298078 | BGI_WZS_WZS973 | Illumina HiSeq 2000 | 23.68 |
| PRJNA213179 | SAMN02298077 | BGI_WZS_WZS955 | Illumina HiSeq 2000 | 23.29 |
| PRJNA213179 | SAMN02298076 | BGI_WZS_WZS947 | Illumina HiSeq 2000 | 22.85 |
| PRJNA213179 | SAMN02298075 | BGI_WZS_WZS889 | Illumina HiSeq 2000 | 22.74 |
| PRJNA213179 | SAMN02298074 | BGI_WZS_WZS809 | Illumina HiSeq 2000 | 23.03 |
| PRJNA213179 | SAMN02298073 | BGI_WZS_WZS149 | Illumina HiSeq 2000 | 23.06 |
| PRJEB1683 | SAMEA1557398 | Xiang (domestic) | Illumina HiSeq 2000 | 9.17 |
| PRJEB1683 | SAMEA1557385 | Xiang (domestic) | Illumina HiSeq 2000 | 8.93 |
| PRJNA186497 | SAMN01894438 | Penzhou pig | Illumina HiSeq 2000 | 5.38 |
| PRJEB9922 | SAMEA3497795 | ASD_Jiangquhai | Illumina HiSeq 2000 | 11.77 |
| PRJEB9922 | SAMEA3497796 | ASD_Jiangquhai | Illumina HiSeq 2000 | 8.70 |
| PRJEB9922 | SAMEA3497797 | ASD_Jiangquhai | Illumina HiSeq 2000 | 9.09 |
| PRJEB9922 | SAMEA3497798 | ASD_Jiangquhai | Illumina HiSeq 2000 | 11.37 |
| PRJEB9922 | SAMEA3497799 | ASD_Jiangquhai | Illumina HiSeq 2000 | 11.85 |
| PRJNA238851 | SAMN02646546 | Tongcheng pigs | Illumina HiSeq 2000 | 6.81 |
| PRJNA238851 | SAMN02646545 | Tongcheng pigs | Illumina HiSeq 2000 | 9.42 |
| PRJNA238851 | SAMN02646544 | Tongcheng pigs | Illumina HiSeq 2000 | 7.56 |
| PRJNA238851 | SAMN02646543 | Tongcheng pigs | Illumina HiSeq 2000 | 9.27 |
| PRJNA238851 | SAMN02646525 | Tongcheng pigs | Illumina HiSeq 2000 | 20.78 |
| PRJNA378496 | SAMN06610393 | Meishan | Illumina HiSeq 2000 | 12.65 |
| PRJNA378496 | SAMN06610205 | Meishan | Illumina HiSeq 2000 | 10.65 |
| PRJNA378496 | SAMN06607518 | Meishan | Illumina HiSeq 2000 | 11.33 |
| PRJNA378496 | SAMN06606917 | Meishan | Illumina HiSeq 2000 | 8.65 |
| PRJNA378496 | SAMN06603627 | Meishan | Illumina HiSeq 2000 | 5.66 |
| PRJNA378496 | SAMN06603626 | Meishan | Illumina HiSeq 2000 | 12.26 |
| PRJNA378496 | SAMN06603524 | Meishan | Illumina HiSeq 2000 | 7.39 |
| PRJNA378496 | SAMN06603407 | Meishan | Illumina HiSeq 2000 | 11.86 |
| PRJNA378496 | SAMN06603369 | Meishan | Illumina HiSeq 2000 | 12.24 |
| PRJNA378496 | SAMN06603321 | Meishan | Illumina HiSeq 2000 | 10.91 |
| PRJNA378496 | SAMN06603310 | Meishan | Illumina HiSeq 2000 | 10.88 |
| PRJNA378496 | SAMN06579320 | Meishan | Illumina HiSeq 2000 | 11.70 |
| PRJNA378496 | SAMN06579197 | Meishan | Illumina HiSeq 2000 | 5.71 |
| PRJNA378496 | SAMN06579151 | Meishan | Illumina HiSeq 2000 | 10.35 |
| PRJNA378496 | SAMN06564163 | Meishan | Illumina HiSeq 2000 | 10.79 |
| PRJNA378496 | SAMN06563661 | Meishan | Illumina HiSeq 2000 | 9.64 |
| PRJNA378496 | SAMN06563583 | Meishan | Illumina HiSeq 2000 | 12.78 |
| PRJNA378496 | SAMN06563369 | Meishan | Illumina HiSeq 2000 | 11.18 |
| PRJNA378496 | SAMN06563284 | Meishan | Illumina HiSeq 2000 | 12.21 |
| PRJNA378496 | SAMN06562984 | Meishan | Illumina HiSeq 2000 | 12.11 |
| PRJNA378496 | SAMN06562978 | Meishan | Illumina HiSeq 2000 | 11.23 |
| PRJNA378496 | SAMN06562977 | Meishan | Illumina HiSeq 2000 | 11.01 |
| PRJNA378496 | SAMN06562971 | Meishan | Illumina HiSeq 2000 | 12.43 |
| PRJNA378496 | SAMN06562959 | Meishan | Illumina HiSeq 2000 | 6.97 |
| PRJNA378496 | SAMN06562956 | Meishan | Illumina HiSeq 2000 | 11.03 |
| PRJNA378496 | SAMN06562602 | Meishan | Illumina HiSeq 2000 | 12.82 |
| PRJNA378496 | SAMN06562589 | Meishan | Illumina HiSeq 2000 | 10.96 |
| PRJNA378496 | SAMN06562584 | Meishan | Illumina HiSeq 2000 | 12.13 |
| PRJNA378496 | SAMN06562578 | Meishan | Illumina HiSeq 2000 | 6.18 |
| PRJNA378496 | SAMN06560428 | Meishan | Illumina HiSeq 2000 | 9.79 |
| PRJNA378496 | SAMN06556517 | Meishan | Illumina HiSeq 2000 | 10.43 |
| PRJNA378496 | SAMN06560458 | Meishan | Illumina HiSeq 2000 | 12.71 |
| PRJNA378496 | SAMN06560373 | Meishan | Illumina HiSeq 2000 | 12.27 |
| PRJNA524263 | SAMN11019718 | Wannan Black pig | HiSeq X Ten | 6.74 |
| PRJNA524263 | SAMN11019717 | Wannan Black pig | HiSeq X Ten | 6.74 |
| PRJNA524263 | SAMN11019716 | Wannan Black pig | HiSeq X Ten | 6.74 |
| PRJNA524263 | SAMN11019715 | Wannan Black pig | HiSeq X Ten | 6.74 |
| PRJNA524263 | SAMN11019714 | Wannan Black pig | HiSeq X Ten | 6.74 |
| PRJNA524263 | SAMN11019713 | Wannan Black pig | HiSeq X Ten | 6.74 |
| PRJNA524263 | SAMN11019712 | Wannan Black pig | HiSeq X Ten | 6.74 |
| PRJCA003970 | SAMC291048 | Pudong Black pig | Illumina HiSeq 2000 | 27.00 |
| PRJCA003970 | SAMC291049 | Pudong Black pig | Illumina HiSeq 2000 | 25.00 |
| PRJCA003970 | SAMC291051 | Pudong Black pig | Illumina HiSeq 2000 | 26.00 |
| PRJCA003970 | SAMC291052 | Pudong Black pig | Illumina HiSeq 2000 | 24.00 |
| PRJNA343658 | SAMN05791674 | Duroc | Illumina HiSeq 2000 | 14.18 |
| PRJNA343658 | SAMN05791673 | Duroc | Illumina HiSeq 2000 | 9.84 |
| PRJNA343658 | SAMN05791672 | Duroc | Illumina HiSeq 2000 | 11.08 |
| PRJNA343658 | SAMN05791671 | Duroc | Illumina HiSeq 2000 | 14.31 |
| PRJNA343658 | SAMN05791670 | Duroc | Illumina HiSeq 2000 | 13.35 |
| PRJNA343658 | SAMN05791667 | Duroc | Illumina HiSeq 2000 | 22.12 |
| PRJNA343658 | SAMN05791666 | Duroc | Illumina HiSeq 2000 | 19.35 |
| PRJNA343658 | SAMN05791665 | Duroc | Illumina HiSeq 2000 | 15.60 |
| PRJNA343658 | SAMN05791664 | Duroc | Illumina HiSeq 2000 | 12.68 |
| PRJNA343658 | SAMN05791661 | Duroc | Illumina HiSeq 2000 | 13.91 |
| PRJNA378496 | SAMN06562649 | Durocs | Illumina HiSeq 2000 | 3.34 |
| PRJNA378496 | SAMN06562603 | Durocs | Illumina HiSeq 2000 | 6.26 |
| PRJNA343658 | SAMN05791662 | Landrace | Illumina HiSeq 2000 | 16.70 |
| PRJNA343658 | SAMN05791659 | Landrace | Illumina HiSeq 2000 | 13.50 |
| PRJNA343658 | SAMN05791657 | Landrace | Illumina HiSeq 2000 | 19.98 |
| PRJNA343658 | SAMN05791656 | Landrace | Illumina HiSeq 2000 | 16.08 |
| PRJNA343658 | SAMN05791655 | Landrace | Illumina HiSeq 2000 | 13.10 |
| PRJNA343658 | SAMN05791653 | Landrace | Illumina HiSeq 2000 | 21.81 |
| PRJNA343658 | SAMN05791652 | Landrace | Illumina HiSeq 2000 | 20.01 |
| PRJNA343658 | SAMN05791651 | Landrace | Illumina HiSeq 2000 | 15.21 |
| PRJNA343658 | SAMN05791650 | Landrace | Illumina HiSeq 2000 | 13.68 |
| PRJNA343658 | SAMN05791649 | Landrace | Illumina HiSeq 2000 | 18.22 |
| PRJNA343658 | SAMN05791648 | Landrace | Illumina HiSeq 2000 | 19.35 |
| PRJEB9922 | SAMEA3497827 | EUD_Berkshire | Illumina HiSeq 2000 | 13.33 |
| PRJEB9922 | SAMEA3497828 | EUD_Berkshire | Illumina HiSeq 2000 | 11.40 |
| PRJEB9922 | SAMEA3497843 | EUD_Berkshire | Illumina HiSeq 2000 | 10.80 |
| PRJEB9922 | SAMEA3497844 | EUD_Berkshire | Illumina HiSeq 2000 | 10.55 |
| PRJEB9922 | SAMEA3497856 | EUD_Middle_White | Illumina HiSeq 2000 | 13.09 |
| PRJEB9922 | SAMEA3497857 | EUD_Middle_White | Illumina HiSeq 2000 | 8.84 |
| PRJEB9326 | SAMEA3376935 | Netherlands | Illumina HiSeq 2000 | 7.52 |
| PRJEB9326 | SAMEA3376936 | Netherlands | Illumina HiSeq 2000 | 11.71 |
| PRJEB9326 | SAMEA3376937 | Netherlands | Illumina HiSeq 2000 | 13.68 |
| PRJEB9326 | SAMEA3376938 | Netherlands | Illumina HiSeq 2000 | 14.13 |
| PRJEB9326 | SAMEA3376939 | Netherlands | Illumina HiSeq 2000 | 14.82 |
| PRJEB9326 | SAMEA3376940 | Netherlands | Illumina HiSeq 2000 | 9.60 |
| PRJEB9326 | SAMEA3376941 | Netherlands | Illumina HiSeq 2000 | 11.26 |
| PRJEB9326 | SAMEA3376942 | Netherlands | Illumina HiSeq 2000 | 11.28 |
| PRJEB9326 | SAMEA3376943 | Netherlands | Illumina HiSeq 2000 | 11.46 |
| PRJEB9326 | SAMEA3376944 | Netherlands | Illumina HiSeq 2000 | 11.95 |
| PRJEB9922 | SAMEA3497829 | EUD_British_Saddleback | Illumina HiSeq 2000 | 8.39 |
| PRJEB9922 | SAMEA3497830 | EUD_British_Saddleback | Illumina HiSeq 2000 | 12.79 |
| PRJEB9922 | SAMEA3497835 | EUD_British_Saddleback | Illumina HiSeq 2000 | 12.06 |
| PRJEB9922 | SAMEA3497836 | EUD_British_Saddleback | Illumina HiSeq 2000 | 9.60 |
| PRJEB9922 | SAMEA3497854 | EUD_Mangalica | Illumina HiSeq 2000 | 10.20 |
| PRJEB9922 | SAMEA3497855 | EUD_Mangalica | Illumina HiSeq 2000 | 11.44 |
| PRJEB1683 | SAMEA1557405 | Landrace (domestic) | Illumina HiSeq 2000 | 10.58 |
| PRJEB1683 | SAMEA1557416 | Landrace (domestic) | Illumina HiSeq 2000 | 11.29 |
| PRJEB1683 | SAMEA1557426 | Landrace (domestic) | Illumina HiSeq 2000 | 8.13 |
| PRJEB1683 | SAMEA1557436 | Landrace (domestic) | Illumina HiSeq 2000 | 8.27 |
| PRJEB1683 | SAMEA1557390 | Landrace (domestic) | Illumina HiSeq 2000 | 8.51 |
| PRJEB1683 | SAMEA1557412 | Large White (domestic) | Illumina HiSeq 2000 | 7.40 |
| PRJEB1683 | SAMEA1557415 | Large White (domestic) | Illumina HiSeq 2000 | 10.47 |
| PRJEB1683 | SAMEA1557431 | Large White (domestic) | Illumina HiSeq 2000 | 10.51 |
| PRJEB1683 | SAMEA1557413 | Large White (domestic) | Illumina HiSeq 2000 | 10.67 |
| PRJEB1683 | SAMEA1557402 | Large White (domestic) | Illumina HiSeq 2000 | 9.92 |


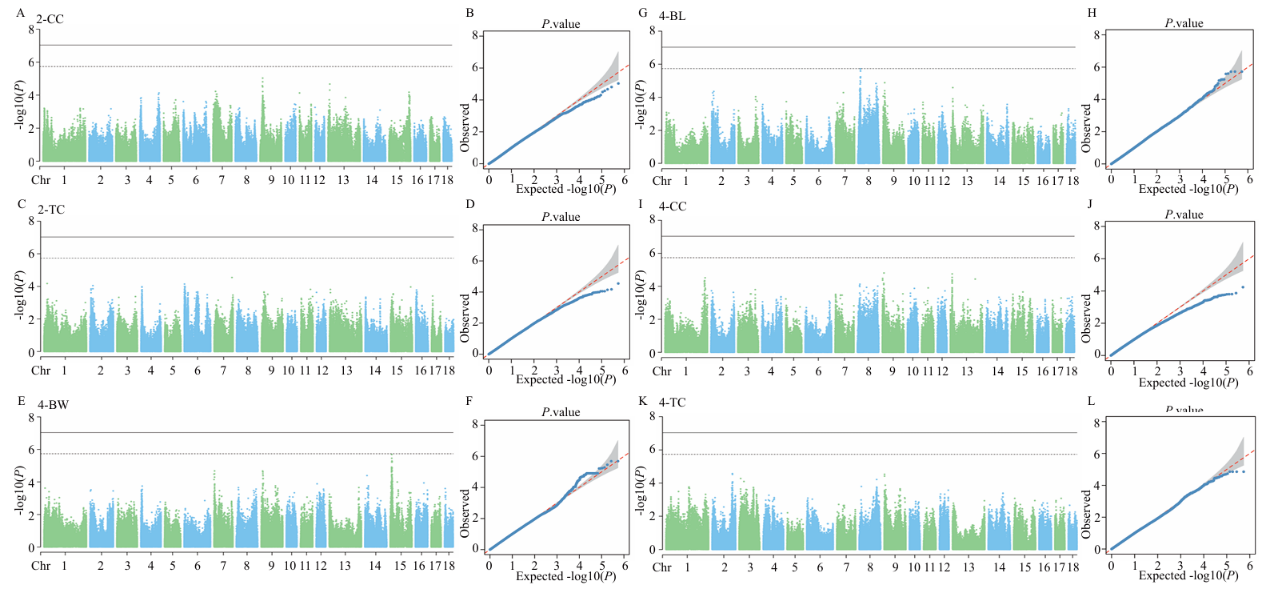

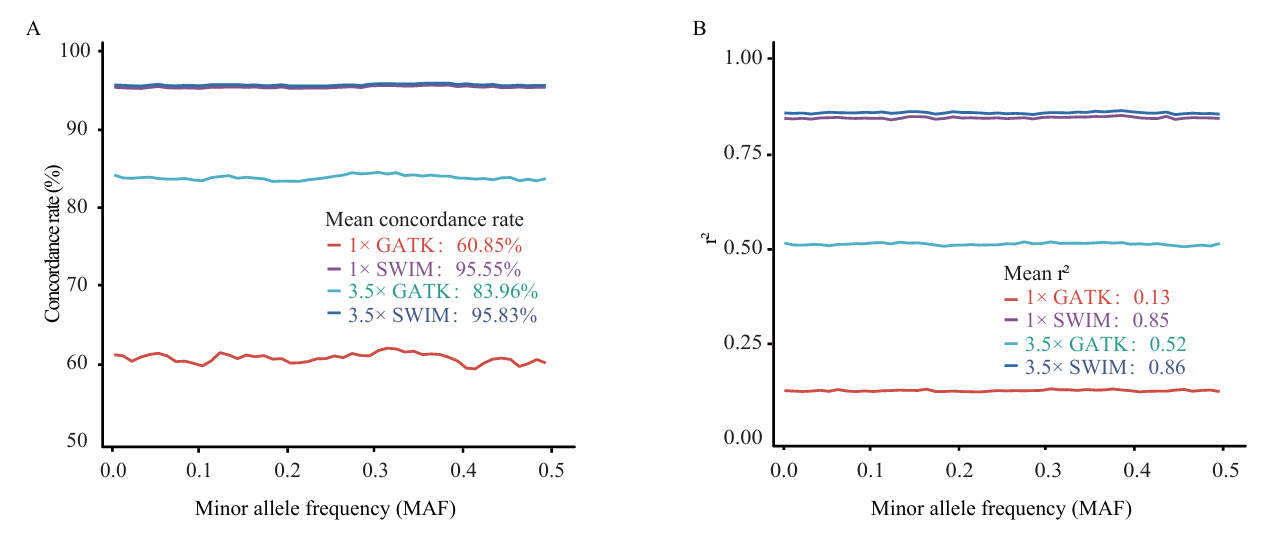
**Fig. S1** Accuracy of imputation in pigs. (A) Concordance rate of imputed versus observed genotypes before and after imputation at different sequencing depth. Mean concordance rate across all variants is also indicated on the plot for each sequencing depth before and after imputation; (B) r^2^ of imputed versus observed genotypes before and after imputation at different sequencing depth. Mean r^2^ across all variants is also indicated on the plot for each sequencing depth before and after imputation.

**Fig. S2.** Manhattan and Q-Q plots of GWAS based on imputation using SWIM for four growth traits. (A, C, E, G, I, K) Manhattan plot for 2-CC, 2-TC, 4-BW, 4-BL, 4-CC, 4-TC; (B, D, F, H) Quantitative-Quantitative (Q-Q) plot for 2-CC, 2-TC, 4-BW, 4-BL, 4-CC, 4-TC; The solid line represents the genome-wide significance level (9.25×10^−8^); The dashed line represents the suggestive significance; Abbreviations: 2-CC = chest circumference at 2 months, 2-TC = tube circumference at 2 months, 4-BW = body weight at 4 months, 4-BL = body length at 4 months, 4-CC = chest circumference at 4 months, 4-TC = tube circumference at 4 months.

**Table S2.** The annotated genes with less than 1 Mb of significant and suggestive significant SNPs

| Months | Traits | Model | Chr^1^ | Position | *P*-Value | Candidate Gene |
| --- | --- | --- | --- | --- | --- | --- |
| 2 | BW^2^ | BLINK | 15 | 129888533 | 1.60E-10 | *LOC100621775 LOC106506390 LOC106508684 SPHKAP PID1* |
| 2 | BW | MLM | 15 | 129888533 | 9.64E-07 | *LOC100621775 LOC106506390 LOC106508684 SPHKAP PID1* |
| 2 | BW | FarmCPU | 15 | 129809430 | 1.82E-06 | *LOC100621775 LOC106506390 LOC106508684 SPHKAP PID1* |
| 2 | BW | FarmCPU | 15 | 129888533 | 5.48E-07 | *LOC100621775 LOC106506390 LOC106508684 SPHKAP PID1* |
| 2 | BL^3^ | FarmCPU | 15 | 132315138 | 1.67E-10 | *B3GNT7 LOC110257014 LOC110256923 LOC106506397 TEX44 LOC106506399 LOC106508574 PTMA LOC110257202 NPPC LOC110257024NCL LOC102158015 NMUR1 LOC106506398 COPS7B PSMD1 HTR2B ARMC9 PDE6D*  *DIS3L2 LOC106506393* |
| 2 | BL | BLINK | 4 | 124246502 | 2.22E-08 | *LOC110260398 RPL5 GFI1 DR1 TMED5 MTF2 LOC110260382*  *RPAP2 CCDC18 FAM69A EVI5*  *LOC100627999 FNBP1L* |
| 2 | BL | BLINK | 18 | 20163486 | 7.15E-08 | *IRF5 LOC100518456 ATP6V1F*  *LOC110257581 TRNAP-AGG-8 OPN1SW HILPDA TRNAE-UUC-48*  *LOC110257533 MIR129B LRRC4*  *KCP LOC110257580 FLNC*  *CCDC136 CALU FAM71F1 IMPDH1*  *PRRT4 LEP TNPO3 RBM28 SND1 FAM71F2* |
| 2 | BL | MLM | 10 | 68503232 | 1.48E-06 | *LOC100625049 LOC102160817 LOC110255731 LOC106505222 LOC106505221 LOC100625534 LOC110255732 WDR37 GTPBP4 LARP4B DIP2C ADARB2* |
| 2 | BL | FarmCPU | 1 | 52055241 | 1.79E-06 | *RIMS1 LOC106508874 KCNQ5* |
| 2 | BL | FarmCPU | 4 | 5817122 | 1.69E-06 | *LOC110260446 LOC110260213 LOC110260214 KHDRBS3* |
| 2 | BL | FarmCPU | 4 | 124246502 | 8.59E-07 | *LOC110260398 RPL5 GFI1 DR1 TMED5 MTF2 LOC110260382 RPAP2 CCDC18 FAM69A EVI5*  *LOC100627999 FNBP1L* |
| 2 | BL | BLINK | 7 | 11352394 | 5.26E-07 | *LOC106504331 LOC110261782*  *LOC110261784 LOC102163473*  *LOC110261781 LOC100739462*  *LOC110261785 LOC106504325 LOC110261780 LOC110261783*  *DTNBP1 LOC110261786 JARID2* |
| 2 | BL | BLINK | 13 | 90418441 | 1.22E-06 | *LOC102163150 LOC110256533*  *SERP1 COMMD2 LOC102162381*  *ANKUB1 LOC110256536 TSC22D2*  *EIF2A LOC102163661 ERICH6*  *LOC102162026 LOC106508818*  *SELENOT RNF13 PFN2* |
| 2 | BH^4^ | FarmCPU | 5 | 70115422 | 6.16E-09 | *LOC110260761 LOC100519429 ATP6V1E1 BCL2L13 PEX26 TUBA8*  *USP18 LOC100737051BID MICAL3 CPNE8* |
| 2 | BH | FarmCPU | 7 | 9879713 | 6.70E-08 | *MIR9837 LOC110261779 LOC110261777 NOL7 RANBP MCUR1 LOC106504323 TBC1D7 GFOD1 SIRT5 RNF182 CD83 PHACTR1* |
| 2 | BH | FarmCPU | 7 | 29282528 | 8.35E-08 | *RPS18 B3GALT4 WDR46 PFDN6 RGL2 ZBTB22 DAXX PHF1 ZBTB9 BEND6 VPS52 TAPBP KIFC1 CUTA*  *SYNGAP1 DST COL21A1* |
| 2 | BH | FarmCPU | 16 | 1154491 | 3.07E-08 | *CTNND2* |
| 2 | BH | FarmCPU | 15 | 41506661 | 1.24E-07 | *LOC110257080 LOC110257063 TENM3* |
| 2 | CC^5^ | BLINK | 5 | 78728345 | 4.83E-09 | *LOC102167685 TMEM106C LOC102162871 LOC100621423*  *LOC100516553 LOC100516910*  *LOC100517280 LOC100155591*  *LOC100156793 LOC110260778*  *LOC100627368 LOC110260592*  *LOC110260593 LOC110260594*  *LOC100737025 LOC100519092*  *LOC100737069 LOC100622380*  *LOC100622479 LOC100622582*  *LOC110260595 LOC102166816*  *LOC100622785 LOC100622090 COL2A1 LOC110260776 SENP1 ASB8 LOC100621110 CCDC184 LOC102161787 ZNF641 LOC100516735 LOC100517094 LOC100517469 C5H12orf54 LOC100737106 LOC100622682 LALBA VDR PFKM LOC106510393* |
| 2 | CC | BLINK | 9 | 16268375 | 1.25E-11 | *LOC110262275 LOC110262273*  *LOC106504825 LOC110262274*  *LOC106504828 LOC102160854* |
| 4 | BW | BLINK | 15 | 9758617 | 6.56E-11 | *LOC110256980 LRP1B* |
| 4 | BH | FarmCPU | 7 | 11211250 | 5.28E-12 | *MIR9807 LOC106504331 LOC110261782 LOC110261784*  *LOC102163473 LOC110261781*  *LOC100739462 LOC106504325*  *LOC110261780 LOC110261783*  *DTNBP1 JARID2* |
| 4 | BH | FarmCPU | 13 | 13335332 | 7.36E-08 | *LOC106505556 LOC110256539*  *LRRC3B NEK10* |
| 4 | BH | BLINK | 7 | 11211250 | 7.36E-08 | *MIR9807 LOC106504331*  *LOC110261782 LOC110261784*  *LOC102163473 LOC110261781*  *LOC100739462 LOC106504325*  *LOC110261780 LOC110261783*  *DTNBP1 JARID2* |
| 4 | BH | BLINK | 13 | 13335332 | 3.22E-08 | *LOC110256539 LRRC3B NEK10* |
| 4 | BH | FarmCPU | 1 | 31679073 | 8.22E-07 | *CTGF LOC102160401 LOC110260060 ENPP1*  *ENPP3 ARG1 LOC106508846 STX7 MOXD1 MED23* |
| 4 | BH | FarmCPU | 7 | 11211250 | 5.28E-12 | *MIR9807 LOC106504331 LOC110261782 LOC110261784 LOC102163473 LOC110261781*  *LOC100739462 LOC106504325*  *LOC110261780 LOC110261783*  *DTNBP1 JARID2* |
| 4 | BH | FarmCPU | 9 | 8996518 | 6.94E-07 | *LOC110255398 LOC106504803*  *LIPT2 LOC102158166*  *LOC110255399 LOC100739258*  *LOC100627340 LOC100739332*  *LOC100739359 C2CD3 PGM2L1*  *LOC106504800 KCNE3 LOC106504801 SPCS2 NEU PPME1*  *P4HA3 POLD3 CHRDL2 RNF169*  *XRRA1 LOC102160367 SLCO2B1* |

^1^Chr: chromosome; ^2^BW: body weight; ^3^BL: body length; ^4^BH: body height; ^5^CC = chest circumference.


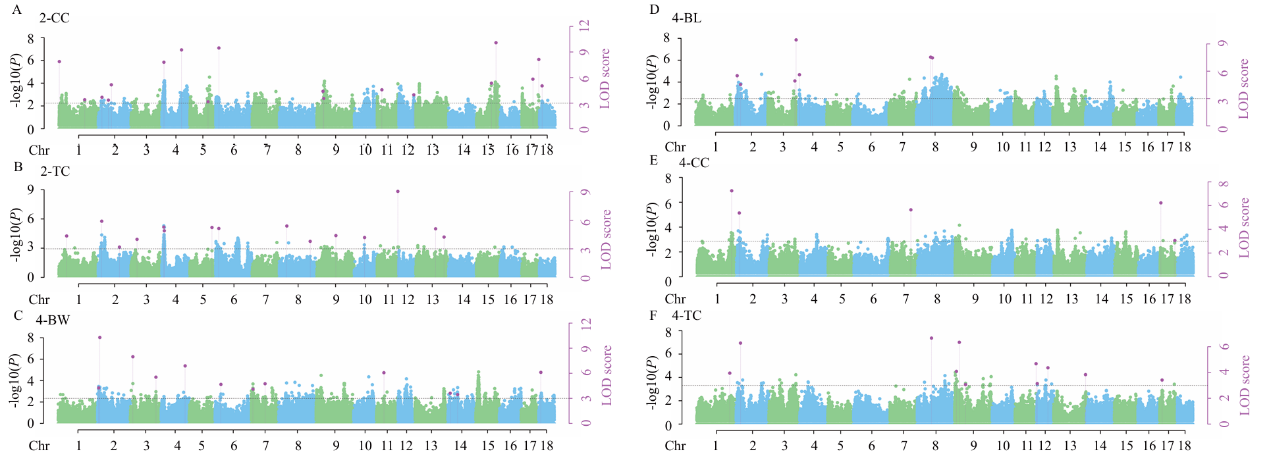
**Fig. S3** Manhattan plots of multi-Locus GWAS based on imputation using SWIM reference panel for four growth traits. (A, B, C, D) Manhattan plots for 2-BW, 2-BL, 2-BH, 4-BH. Manhattan plots indicate LOD scores for genome-wide SNPs (y-axis) plotted against their respective positions on each chromosome (x-axis), and the horizontal lines indicate the thresholds for significance (LOD score = 3). Abbreviations: 2-CC = chest circumference at 2 months, 2-TC = tube circumference at 2 months, 4-BW = body weight at 4 months, 4-BL = body length at 4 months, 4-CC = chest circumference at 4 months, 4-TC = tube circumference at 4 months.

**Table S3.** Description of significant SNPs identified by multi-locus methods as associated with growth traits

| Traits | SNP^1^ | Chr^2^ | Position | LOD score | r^2^(%)^3^ | Candidate Gene |
| --- | --- | --- | --- | --- | --- | --- |
| 2-CC^4^ | 1:1435781 | 1 | 1435781 | 7.8744 | 3.9767 | *FRMD1* |
| 2-CC | 1:151212336 | 1 | 151212336 | 3.3985 | 1.0607 | *CBLN2* |
| 2-CC | 2:10667280 | 2 | 10667280 | 3.6831 | 1.1329 | *PRPF19* |
| 2-CC | 2:35756235 | 2 | 35756235 | 3.3559 | 1.1072 | *LUZP2* |
| 2-CC | 2:54392449 | 2 | 54392449 | 5.1511 | 1.9152 | *-* |
| 2-CC | 4:5824234 | 4 | 5824234 | 7.7967 | 4.2397 | *KHDRBS3* |
| 2-CC | 4:111813556 | 4 | 111813556 | 9.2463 | 4.7591 | *FAM102B* |
| 2-CC | 5:74306116 | 5 | 74306116 | 3.1628 | 0.7689 | *PUS7L* |
| 2-CC | 6:4362106 | 6 | 4362106 | 9.4708 | 4.6595 | *HSBP1* |
| 2-CC | 9:13267496 | 9 | 13267496 | 4.3867 | 1.6816 | *TENM4* |
| 2-CC | 9:14507015 | 9 | 14507015 | 3.5485 | 1.1614 | *-* |
| 2-CC | 11:14758203 | 11 | 14758203 | 4.5634 | 1.8322 | *NHLRC3* |
| 2-CC | 12:57011143 | 12 | 57011143 | 3.9705 | 1.5358 | *MAP2K4* |
| 2-CC | 15:132340482 | 15 | 132340482 | 10.0853 | 5.0437 | *NPPC* |
| 2-CC | 15:119882549 | 15 | 119882549 | 5.3404 | 2.4013 | *TMBIM1* |
| 2-CC | 17:45388018 | 17 | 45388018 | 5.812 | 3.0745 | *PTPRT* |
| 2-CC | 17:60616836 | 17 | 60616836 | 8.1247 | 3.6599 | *CDH4* |
| 2-CC | 18:7448271 | 18 | 7448271 | 5.0265 | 2.1123 | *TMEM139* |
| 2-TC^5^ | 1:28729081 | 1 | 28729081 | 4.3488 | 2.319 | *PDE7B* |
| 2-TC | 2:10001426 | 2 | 10001426 | 5.9005 | 3.7777 | *TMEM138* |
| 2-TC | 2:118187896 | 2 | 118187896 | 3.18 | 0.8587 | *KCNN2* |
| 2-TC | 3:16874769 | 3 | 16874769 | 3.9949 | 3.528 | *SLC5A2* |
| 2-TC | 4:5824234 | 4 | 5824234 | 5.2814 | 4.1169 | *KHDRBS3* |
| 2-TC | 4:7274704 | 4 | 7274704 | 4.8967 | 2.2561 | *ZFAT* |
| 2-TC | 5:89508241 | 5 | 89508241 | 5.2394 | 2.5936 | *UBE2N* |
| 2-TC | 6:4362106 | 6 | 4362106 | 5.1364 | 2.3106 | *USP10* |
| 2-TC | 8:14485953 | 8 | 14485953 | 5.395 | 2.8558 | *SLIT2* |
| 2-TC | 8:122791009 | 8 | 122791009 | 3.7818 | 1.6567 | *STPG2* |
| 2-TC | 9:55318397 | 9 | 55318397 | 4.3888 | 3.2586 | *KCNJ1* |
| 2-TC | 10:24940817 | 10 | 24940817 | 4.1855 | 1.5145 | *KDM5B* |
| 2-TC | 11:76347156 | 11 | 76347156 | 9.0307 | 7.338 | *MYO16* |
| 2-TC | 13:100900541 | 13 | 100900541 | 5.1024 | 2.6021 | *SPTSSB* |
| 2-TC | 13:189383591 | 13 | 189383591 | 4.2349 | 1.4391 | *GABPA* |
| 4-BW^6^ | 2:4014143 | 2 | 4014143 | 4.2509 | 1.7213 | *CCND1* |
| 4-BW | 2:6923710 | 2 | 6923710 | 10.2846 | 8.547 | *MUS81* |
| 4-BW | 3:5798470 | 3 | 5798470 | 7.9817 | 5.8837 | *TMEM130* |
| 4-BW | 3:112379490 | 3 | 112379490 | 5.5368 | 3.3553 | *SLC5A6* |
| 4-BW | 4:123251466 | 4 | 123251466 | 6.8861 | 3.8455 | *TRNAR* |
| 4-BW | 6:8735738 | 6 | 8735738 | 4.6705 | 2.941 | *WWOX* |
| 4-BW | 7:3520173 | 7 | 3520173 | 4.1047 | 3.7187 | *RPP40* |
| 4-BW | 7:45181609 | 7 | 45181609 | 4.7507 | 2.8208 | *PKHD1* |
| 4-BW | 11:23635602 | 11 | 23635602 | 6.0695 | 3.5778 | *LACC1* |
| 4-BW | 14:4123054 | 14 | 4123054 | 3.6138 | 1.3713 | *SLC18A1* |
| 4-BW | 14:22875633 | 14 | 22875633 | 3.4512 | 1.6876 | *LRCOL1* |
| 4-BW | 18:5449900 | 18 | 5449900 | 6.1099 | 4.5623 | *WDR86* |
| 4-BL^7^ | 2:6269290 | 2 | 6269290 | 5.5057 | 4.6331 | *CCDC87* |
| 4-BL | 2:14548190 | 2 | 14548190 | 4.5401 | 8.2548 | *FAM180B* |
| 4-BL | 3:117344394 | 3 | 117344394 | 4.9335 | 5.8923 | *RHOB* |
| 4-BL | 3:121659940 | 3 | 121659940 | 9.4406 | 12.5652 | *MYCN* |
| 4-BL | 4:3179544 | 4 | 3179544 | 5.6159 | 6.0684 | *KCNK9* |
| 4-BL | 8:34599614 | 8 | 34599614 | 7.5416 | 7.2209 | *KCTD8* |
| 4-BL | 8:40982852 | 8 | 40982852 | 7.4648 | 11.3958 | *PDGFRA* |
| 4-CC^8^ | 1:256763175 | 1 | 256763175 | 7.2449 | 12.3629 | *ASTN2* |
| 4-CC | 2:8090645 | 2 | 8090645 | 5.3761 | 8.1993 | *STIP1* |
| 4-CC | 7:91902493 | 7 | 91902493 | 5.6414 | 9.7021 | *RDH12* |
| 4-CC | 17:1790221 | 17 | 1790221 | 6.2277 | 10.034 | *DLC1* |
| 4-CC | 17:56495183 | 17 | 56495183 | 3.0595 | 4.4564 | *FAM210B* |
| 4-TC^9^ | 1:250868707 | 1 | 250868707 | 3.9507 | 3.0809 | *PTPN3* |
| 4-TC | 2:12079884 | 2 | 12079884 | 6.279 | 3.5482 | *PATL1* |
| 4-TC | 8:34327707 | 8 | 34327707 | 6.6654 | 4.5901 | *KCTD8* |
| 4-TC | 9:4909203 | 9 | 4909203 | 4.0851 | 1.8002 | *HBE1* |
| 4-TC | 9:10607642 | 9 | 10607642 | 6.3402 | 5.5025 | *LRRC32* |
| 4-TC | 9:24531979 | 9 | 24531979 | 3.0625 | 1.8553 | *FAT3* |
| 4-TC | 11:77089747 | 11 | 77089747 | 4.679 | 3.2372 | *SPACA7* |
| 4-TC | 12:4875389 | 12 | 4875389 | 3.1397 | 1.6682 | *RNF157* |
| 4-TC | 12:43261975 | 12 | 43261975 | 4.3622 | 4.7627 | *ADAP2* |
| 4-TC | 13:205452492 | 13 | 205452492 | 3.8326 | 0.9523 | *SLC37A1* |
| 4-TC | 17:6425732 | 17 | 6425732 | 3.4128 | 2.5735 | *-* |

**Table S5.** The significant KEGG pathways of differentially expressed genes

| Entry | ID | *P*-Value | Differentially Expressed Gene |
| --- | --- | --- | --- |
| MAPK signaling pathway | KEGG:04010 | 5.73E-05 | *MAP3K2、ERBB4、DUSP8* |
| Regulation of actin cytoskeleton | KEGG:04810 | 0.003987 | *RAC1、ACTN4、PIP5K1C* |
| cAMP signaling pathway | KEGG:04024 | 0.003987 | *RAC1、ATP1A4、NFATC1* |
| Metabolic pathways | KEGG:01100 | 0.005057 | *AHCY、GUCY1A2、PIK3C2A* |
| Calcium signaling pathway | KEGG:04020 | 0.006589 | *ERBB4、KDR* |
| Mineral absorption | KEGG:04978 | 0.008838 | *TRPM7、ATP1A4、SLC30A1* |
| AMPK signaling pathway | KEGG:04152 | 0.024378 | *PPP2CB、IGF1、PFKFB3* |
| Protein digestion and absorption | KEGG:04974 | 0.027129 | *SLC3A1、COL11A1* |


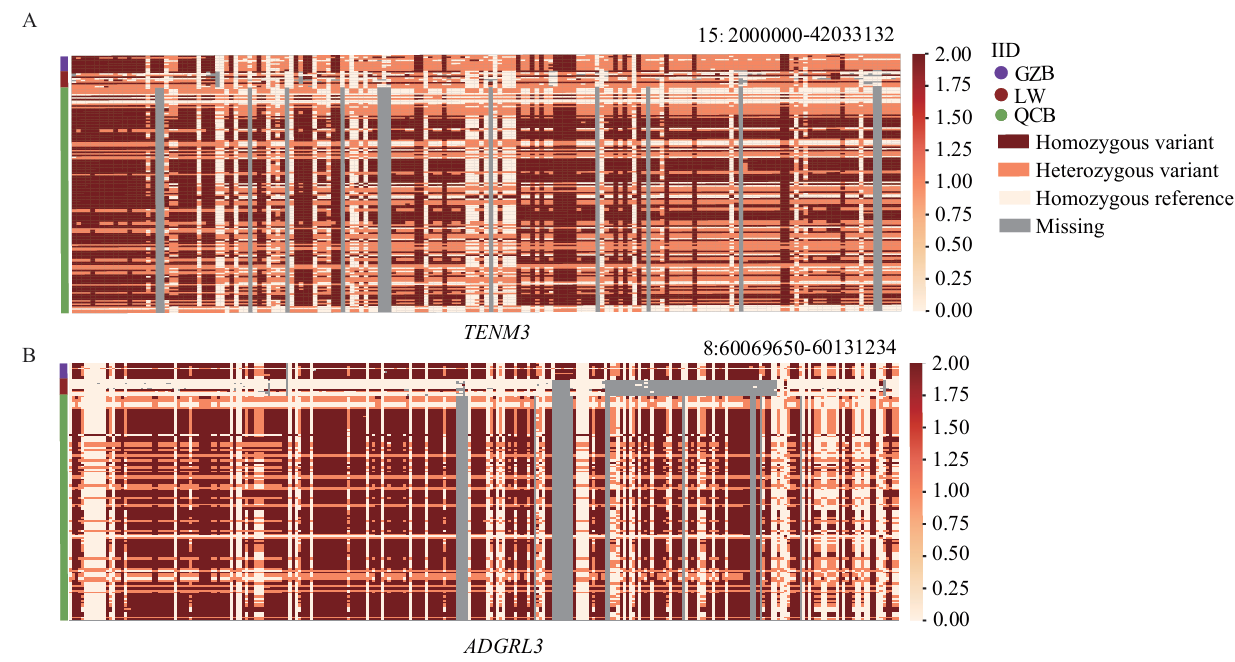
**Fig. S4** Identification of the candidate genes. (A) The genotype of the *TENM3* gene; (B) The genotype of the *ADGRL3* gene.
